# Heterogeneous Tempo and Mode of Conserved Noncoding Sequence Evolution among Four Mammalian Orders

**DOI:** 10.1093/gbe/evt177

**Published:** 2013-11-20

**Authors:** Isaac Adeyemi Babarinde, Naruya Saitou

**Affiliations:** ^1^Department of Genetics, School of Life Science, The Graduate University for Advanced Studies (SOKENDAI), Mishima Japan; ^2^Division of Population Genetics, National Institute of Genetics, Mishima Japan; ^3^Department of Biological Sciences, Graduate School of Science, University of Tokyo, Tokyo Japan

**Keywords:** conserved noncoding sequences, mammals, primates, rodents, carnivores, cetartiodactyls

## Abstract

Conserved noncoding sequences (CNSs) of vertebrates are considered to be closely linked with protein-coding gene regulatory functions. We examined the abundance and genomic distribution of CNSs in four mammalian orders: primates, rodents, carnivores, and cetartiodactyls. We defined the two thresholds for CNS using conservation level of coding genes; using all the three coding positions and using only first and second codon positions. The abundance of CNSs varied among lineages, with primates and rodents having highest and lowest number of CNSs, respectively, whereas carnivores and cetartiodactyls had intermediate values. These CNSs cover 1.3–5.5% of the mammalian genomes and have signatures of selective constraints that are stronger in more ancestral than the recent ones. Evolution of new CNSs as well as retention of ancestral CNSs contribute to the differences in abundance. The genomic distribution of CNSs is dynamic with higher proportions of rodent and primate CNSs located in the introns compared with carnivores and cetartiodactyls. In fact, 19% of orthologous single-copy CNSs between human and dog are located in different genomic regions. If CNSs can be considered as candidates of gene expression regulatory sequences, heterogeneity of CNSs among the four mammalian orders may have played an important role in creating the order-specific phenotypes. Fewer CNSs in rodents suggest that rodent diversity is related to lower regulatory conservation. With CNSs shown to cluster around genes involved in nervous systems and the higher number of primate CNSs, our result suggests that CNSs may be involved in the higher complexity of the primate nervous system.

## Introduction

Conserved noncoding sequence (CNS) analyses have been proved to be computationally powerful in the detection of regulatory elements (Hardison 2000; Levy et al. 2001). Although some noncoding messenger RNA (mRNA) sequences have been found to be conserved, Hemberg et al. (2012) reported that it is four times more likely that a conserved noncoding island is a regulatory element than a noncoding mRNA. ChIP-seq has been reported to be accurate in predicting enhancer activity (Visel et al. 2009). Schmidt et al. (2010), focusing on two transcription factors in liver tissues of five vertebrate species for ChIP-seq analysis, reported that very few regulatory elements are shared by all the species used. However, a mouse ChIP-seq study that examined five transcription factors in 19 tissues and cell types of mouse shows that more than 70% of CNSs function in gene regulation (Shen et al. 2012).

These reports suggest that CNS functions could be specific to tissue, cell type, transcription factor, and/or species. In fact, Shen et al. (2012) clearly demonstrated that the regulatory elements recovered increases with number of tissues and cell types. Some regulatory elements are not conserved (Schmidt et al. 2010), whereas others are conserved. Therefore, the analysis of CNSs should give an idea of the shared regulatory elements. Although Meader et al. (2010) reported high turnover in mammalian functional sequences, many of CNSs are conserved over long evolutionary time (Woolfe et al. 2004) and some are even more conserved than the coding regions (Bejerano et al. 2004; Katzman et al. 2007; Takahashi and Saitou 2012). This is in concordance with the result of several studies that both conserved and nonconserved regions can function as regulatory elements (Chen et al. 2008; McGaughey et al. 2009; Schmidt et al. 2010; Shen et al. 2012).

These reports showed that although some important gene regulations are indispensable and hence conserved, some are experiencing higher turnover rates and thus they are less conserved over a long evolutionary time. Previous reports have shown that arrays of ultraconserved noncoding regions span through the key developmental genes in vertebrate genomes, and those ultraconserved regions have strong positive positional correlation with genes encoding transcription factors (Sandelin et al. 2004; Woolfe et al. 2004). Therefore, CNSs conserved in all members of a lineage are functionally important for the lineage. Among the lineage shared CNSs, a subset of CNSs that are unique to the lineage might be functionally important for the lineage-specific features. Such CNSs that are conserved or lost in an order but not in any other outgroup might hold the key to explain the phenotypic diversity among orders.

It is possible that two species would have alignable sequences, not because the sequences are under functional constraint, but because they diverged recently. One of the important tasks in CNS analyses is setting the appropriate thresholds. It is critical to differentiate sequences that are under real selective constraints from those that have simply not had enough time to accumulate enough mutations that will make them distinguishable. For example, more than 95% of human genomes can be aligned to the chimpanzee genome of which only about 10% has been reported to be under selective constraints (Meader et al. 2010; Ponting and Hardison 2011) although the ENCODE Project Consortium (2012) reported a much higher proportion (80%) of biochemically functional elements in the human genome (see Graur et al. 2013 on the ENCODE paper). One way to handle this task is to use more distantly related species such as human and fugu (Woolfe et al. 2005) or elephant shark and some other vertebrates (Lee et al. 2011). With more distantly related species, any sequence conserved over such a long evolutionary time must be functionally important. The other option is to set a threshold that will filter off hits that are not under functional constraints. In setting the threshold, length and percent identity are usually considered. For example, ultraconserved elements are 100% identical over at least 200 bp length among human, mouse, and rat genomes (Bejerano et al. 2004). Some of the other criteria that have been used are 70% over 100 bp (Duret et al. 1993; Lee et al. 2011), 95% over 50 bp (Sandelin et al. 2004), 95% over 500 bp (Janes et al. 2011), and 98% over 100 bp (Takahashi and Saitou 2012). In this option, however, we have to take cognizance of the differential evolutionary rates and the divergence time of the species. Species with higher substitution rate and those who diverged earlier would have lower percent identity.

Takahashi and Saitou (2012) previously compared CNSs of primates (human and marmoset) and rodents (mouse and rat) and found various differences on CNSs and their flanking protein-coding genes. The stringency of the threshold ensured that only sequences with extremely high selectively constraints are studied. However, functional elements that are not highly conserved would be excluded. In this study, using less stringent but reasonable thresholds, we compared genome sequences of five primate species, three rodent species, three carnivore species, and three cetartiodactyl species. We adjusted for the differential evolutionary rate and divergence time to define CNSs that are under functional constraint. We defined a CNS of one mammalian order as a noncoding part of the genome with at least 100 pb length and the percent divergence similar to or higher than that of protein-coding genes of that order. In setting divergence thresholds, we used whole-coding gene sequences as well as third codon-skipped coding region sequences. These criteria are different from Takahashi and Saitou (2012) who used 98% identity over 100 bp. We discovered that the tempo and mode of CNS evolution differed from order to order among mammals and that recent and more ancestral CNSs are under different constraints.

## Materials and Methods

### Homology Search

The repeat-masked genomes of 24 species were retrieved from the Ensembl genome database, except the sheep genome (*oviAri1*) that was retrieved from the UCSC genome. The species used are listed in supplementary table S1, Supplementary Material online, and their phylogenetic relationship is shown in supplementary figure S1, Supplementary Material online. The genomic coding coordinates were retrieved from Ensembl biomart and UCSC table browser. For most of the species, the genome coverage is at least 6×. To ensure unbiased comparison, we selected the species such that the most distantly related species within every lineage diverged from the reference species 50–60Ma. The coding regions of all the species were masked. We focused on four different mammalian orders: Primates, Rodentia, Carnivora, and Cetartiodactyla. For each order, a reference species was selected based on the quality and availability of genome information. Human, mouse, dog, and cow were used as reference genomes for primates, rodents, carnivores, and cetartiodactyls, respectively.

After masking the genes in each genome, we searched for the sequences that are conserved in each member of a lineage, using the reference genome as the query. BlastN 2.2.25+ (Altschul et al. 1997) was used for whole genome pairwise homology search. The thresholds used were e value of 10^−^^5^ and the database size of 3 × 10^9^. Nonchromosomal sequences (such as mitochondrial DNA, unmapped DNA, and variant DNA) were not included. If two hits are completely overlapping, the shortest hit is discarded. The threshold percent divergences were set for each group (see details later). Hits above the threshold were first retrieved in as much as they are at least 100 bp long. For the remaining hits, we searched for the core in the alignment with highly conserved regions of at least the threshold percent identity and 100 bp length by using sliding windows. This procedure ensures that only alignments of at least the threshold percent identity and 100 bp long in the pairwise search between the reference genome and other members of the group were retained. These conserved sequences are expected to be under functional constraint. Regions of the reference genome that are conserved in all the members of the lineage are potential “group-common” CNSs. Regions of any CNS that overlap RNA gene, pseudogene, or the region that contains masked region is discarded. The resulting CNSs, irrespective of their presence in other species, are referred to as group-common CNSs. We then searched all other species to examine whether these group-common CNSs of one group have homologous sequences or not. By discarding regions conserved in nonmembers, we detected regions that are unique to that group if they are at least 100 bp long. CNSs thus obtained are common to all members of the group but not found in any nongroup species. We refer to these as “group-unique” CNSs.

### Setting the Percent Identity Threshold

It is important to differentiate between homologous regions that are under functional constraint and those that are similar because they have not had enough time to accumulate enough mutation to distinguish the sequences. This is especially important because our study is on lineage-specific CNSs that diverged around 50–60 Ma. As protein-coding genes are under functional constraint, we decided to use gene-based approach to set the threshold. We did not use protein percent identity because our analysis is nucleotide based. We first considered using nonsynonymous substitution and obtained the values for genes with one-to-one correspondence between the reference species and the most diverged species from Ensembl biomart. However, the standard deviation (SD) for nonsynonymous substitutions is very large (see supplementary table S2, Supplementary Material online) with some genes having very high values. We then considered setting the threshold using coding sequences themselves. We retrieved the cDNA sequences of one-to-one (with one-to-one correspondence in Ensembl biomart) orthologous protein-coding genes for the reference and most diverged species of each group from Ensembl biomart (Vilella et al. 2009). We used the longest transcript for each gene in each species. For this estimation, we used human and marmoset for primates, mouse and guinea pig for rodents, dog and cat for carnivores, and cow and pig for cetartiodactyls (see supplementary fig. S1, Supplementary Material online). We used BlastN search to calculate the percent identity of the nucleotide sequences in the two species. If there is more than one local alignment in a gene pair, we used the most conserved alignment. Because of the e-value threshold in the homology search, poorly conserved alignments are filtered off. This filtering results in lower SD.

Nucleotide divergences of the coding regions were normally distributed (*P* < 10^−^^10^) in all lineages. The test for normality is an omnibus test that combines skew and kurtosis test using Scipy package (Jones et al. 2011). The mean divergence value was significantly lower than the mean value of synonymous substitutions and the genomic average (*t*-test *P* < 10^−^^20^) but higher than the mean value of nonsynonymous substitutions (*t*-test *P* < 10^−^^15^) in all lineages. This is reasonable because although most synonymous sites are evolving neutrally, nonsynonymous sites are mostly under selective constraint. We also compared the mean divergence value of the coding regions with the average divergence of all gene and repeat-masked noncoding regions. To do this, we did homology search using BlastN with the gene and repeat-masked genome of the reference species as the query and most diverged species as the subject using the threshold e-value of 10^−^^5^. We used only regions with no duplicates in any of the species. The mean divergence value of the coding regions was significantly lower than the average genomic noncoding divergence (*t*-test *P* = 0) in each lineage. Finally, we considered the proportion of the mean divergence value of the coding regions to the nonsynonymous substitutions (see supplementary table S2, Supplementary Material online). This value is similar in each lineage (∼0.06), suggesting that the mean divergence value of the coding regions is a reasonable threshold. We therefore focused on CNSs that have at most the mean divergence value of the protein-coding genes of the most diverged species to the reference genome. We subsequently refer to this threshold as “whole coding threshold.”

As majority of the third codon sites are synonymous, we decide to set more stringent thresholds using the alignment of coding sequences without using third codons. We obtained the coding regions for the reference genome and the most diverged species for each lineage. We removed bases on third codon positions and concatenated the remaining sequences. We then aligned the concatenated third codon-skipped sequences. The means of percent divergence for each lineage were used as thresholds. These are subsequently referred to as “skip3 thresholds.” Skip3 thresholds are therefore more stringent than whole coding thresholds (supplementary table S3, Supplementary Material online). For each threshold, only sequences with at least 100 bp are considered. It should be noted that these criteria are different from that of Takahashi and Saitou (2012).

### Retention of Ancestral CNSs

The abundance of CNSs in a group is partly a result of the retention and loss of ancestral CNSs. This might be an important force in lineage-specific evolution. To study the retention of ancestral CNSs, we used chicken as a basal species. This is because birds have been reported to have sequences closer to the ancestral genome of amniotes (Bourque et al. 2005). We did independent homology search for genomes of human, mouse, dog, cow, African elephant, opossum, and platypus, with the chicken genome as query. Portions of hits overlapping any known gene were discarded. Using the whole coding and the skip3 thresholds (see supplementary table S3, Supplementary Material online) for sequences with at least 100 bp length, we obtained the pairwise CNSs between chicken and each of the seven species. To obtain the total picture of amniote ancestral CNSs, we made a union set of all the CNSs found in all the species used by merging overlapping hits. These CNSs are the total amniotic ancestral CNSs that are still retained in chicken. We then found CNSs lost at each phylogenetic branch starting from the ancestral CNSs.

To investigate the dynamics of more recent CNSs (those found in eutherian mammal common ancestor), we used the genome sequences of African elephant, which is the immediate outgroup species in our analysis as the reference genome, and searched for CNSs in human, mouse, cow, and dog as representative species for each order. Use of African elephant gave more CNSs because many CNSs that evolved after the split of chicken and mammals are included. We repeated the procedure above to obtain the number of CNSs lost in each lineage.

### Phylogenetic Tree Reconstruction

We extracted tetrapod common CNSs in all the 24 species using whole coding thresholds. For each CNS, we got all the orthologs in all the species and aligned the CNSs using ClustalW (Larkin et al. 2007). These alignments were concatenated and blocks with gaps were removed. A Neighbor-Joining tree (Saitou and Nei 1987) was constructed using MEGA version 5 (Tamura et al. 2011).

### Conservation Levels and Guanine–Cytosine Contents of Flanking Regions of CNSs

We extracted CNSs together with 1,500-bp upstream and downstream flanking sequences and then aligned the sequences using BlastN. For each alignment, we made sliding windows of 50 bp and a step size of 20 bp starting from 30 bp inside the CNSs and calculated the percent identity in each window. We then calculated the average of the mean of the percent identity for each window. We also calculated the average percent identity of 100 bp in the center of the CNSs. For computation of guanine–cytosine (GC) contents, we similarly obtained CNSs with the 1,500-bp upstream and downstream. We made sliding windows of 200-bp and 10-bp step sizes, starting from 50 bp into the CNSs for calculating the mean GC content values for each window.

### Single Nucleotide Polymorphism and Derived Allele Frequency Analyses

We downloaded human single nucleotide polymorphism (SNP 135) database from Ensembl site. We used all SNPs as well as the single nucleotide variants (SNVs) and found the coverage of SNPs in CNSs and random sequences. For derived allele frequency (DAF) analysis, we retrieved hapmap SNP frequency data of Yoruba population in Ibadan, Nigeria, from UCSC table browser. The ancestral alleles of SNPs overlapping the CNSs or random sequences were determined using chimpanzee sequences.

### Gene Ontology Analysis

We used a modified form of closest gene model for the gene ontology analysis. For a CNS to regulate the closest gene in a reference species, we assumed that in another species, the ortholog of the gene must be the closest to the putative ortholog of the CNS. We downloaded orthologous genes for the reference species and the most distantly related species in the group from Ensembl biomart (Vilella et al. 2009). For each group-unique CNS, we retrieved the list of genes found 1 Mbp upstream and downstream. Lettice et al. (2003) and El-Kasti et al. (2012) reported 1-Mbp regulation and Vavouri et al. (2005) reported that focusing on 1 Mb range is suitable to obtain the likely target genes. We matched the orthologous genes to each homologous CNS pair and calculated the average distance, defined as the sum of the gene-CNS distance in species 1 and gene-CNS distance in species 2 divided by 2. The orthologs with the shortest average distance are considered as the likely target genes for the homologous CNSs. We checked the functional classification of the tetrapod common CNS-associated genes using PANTHER 7.0 software (Thomas et al. 2003). Because of the limited gene number in the PANTHER database, we manually checked the enrichment of genes using the binomial test as described in PANTHER. Statistical analyses were done using R (R Core Team 2013) and Scipy (Jones et al. 2011).

### Genomic Distribution

We downloaded gene coordinates as well as the corresponding 5′ untranslated region (UTR) and 3′ UTR coordinates for the reference species from Ensembl Biomart. For this analysis, we rely almost exclusively on Ensembl gene annotations. Although we used Ensembl build 66 for other species, we used Ensembl build 70 for dog because the gene annotation of dog has recently been improved using cDNA sequences from several tissues. We extracted the promoter coordinates from the gene coordinates. We define promoter region as the region within 1,000-bp upstream of transcription start site. We then found CNSs that are located on UTR, promoter, intronic, and intergenic regions. To see whether the genomic location of orthologous CNSs is always constant, we first searched whether any of the CNSs has duplicates in the whole genome. Because CNSs with duplicates may have more than one genomic position, we excluded all the CNSs with duplicates in any of the four reference species. We then mapped the genomic location of single-copy orthologous CNSs in each species.

To have the overall picture of the distribution of CNSs and genes in a chromosome, we used sliding windows of 1 Mbp size and step size of 100 kbp. To investigate whether there is a correlation between gene density and CNS density, we counted the number of CNSs and genes in each window. We calculated the Pearson correlation coefficient between CNSs and genes for all the bins with at least one CNS and at least one gene. We used only windows with at least a gene and a CNS because some windows, especially, around the centromeres do not have any gene or CNS.

Unless otherwise stated, all scripts used for these analyses were written by one of us (I.A.B.) using Python and are available upon request. All coordinates of the CNSs are also available upon request.

## Results

### Lineage Common CNSs

**Fig. 1.—:**
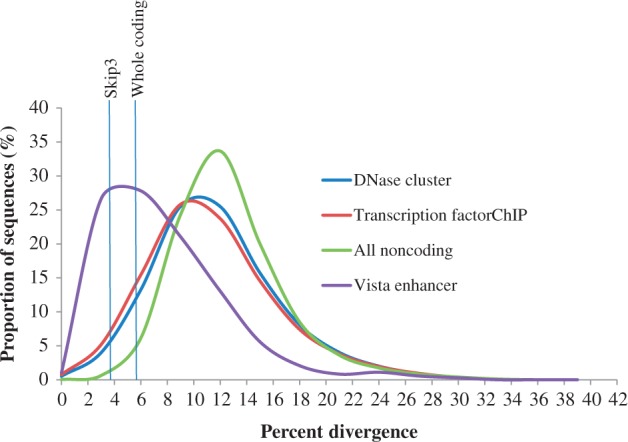
Assessing the suitability of percent divergence thresholds. The two thresholds are comparable to the divergence of vista enhancer elements and higher than the average of all alignable noncoding sequences.

Our analysis takes lineage differential evolutionary rates into consideration (see Materials and Methods). The results using the whole coding threshold and the skip3 threshold are presented in supplementary table S3, Supplementary Material online. Rodents have a significantly high percent difference and primates have the lowest percent difference (*t*-test *P* < 10^−^^180^), consistent with Li et al. (1996) and several other studies that rodents have higher substitution rates. Many experimentally verified functional elements, such as vista enhancer elements and transcription factor ChIP sequences, have been reported for human. Vista enhancers are highly conserved noncoding regions that have been experimentally confirmed to have regulatory function. Also, DNase clusters have been reported to have regulatory signatures (Crawford et al. 2006). To examine the suitability of our thresholds, we obtained the human sequences of these elements from UCSC table browser. Using BlastN, we searched for the homologous marmoset sequence and calculated the percent divergence of each element. We then found the distributions of the percent divergence between these human elements and the corresponding marmoset orthologs. Our analysis shows that our thresholds in primates are reasonable (fig. 1). The thresholds are close to the peak of vista enhancers. As the same procedure was used for the four lineages, we assumed that our thresholds are generally reasonable. Furthermore, considering the average genomic noncoding divergence (see supplementary table S2, Supplementary Material online), alignments of our whole coding threshold divergence levels are less likely to be observed by chance (binomial *P* < 0.05) in all lineages. The chance is even less likely under skip3 threshold (binomial *P* < 0.005; see supplementary table S3, Supplementary Material online). Using the whole coding threshold and the skip3 threshold, as well as minimum length of 100 bp, we searched for the lineage common CNSs. They include CNSs that originated before the emergence or in the common ancestor of each lineage. Primates have the highest number of common CNSs (861,183) compared with 148,848, 491,078, and 257,051 CNSs in rodents, carnivores, and cetartiodactyls, respectively, using the whole coding threshold. The lineage common CNSs covered 5.52%, 1.28%, 2.14%, and 2.12% of human, mouse, dog, and cow chromosome-mapped genomes, respectively. When more stringent skip3 thresholds were used, the numbers of CNSs retrieved were 323,351, 62,985, 220,009, and 130,381 for primates, rodents, carnivores, and cetartiodactyls, respectively (see supplementary fig. S2, Supplementary Material online). It is important to note here that five primate species were used compared with three each in other lineages (see supplementary fig. S1, Supplementary Material online). Because of higher turnover rate of CNSs (Meader et al. 2010) and high independent losses of conserved noncoding DNA elements (CNEs; Hiller et al. 2012), lineages with higher number of species should have fewer CNSs. However, primate CNSs are more than 5-fold that of rodents and about 3-fold that of cetartiodactyls. The carnivore CNSs are about twice that of cetartiodactyls and 3-fold that of rodents despite similar genome sizes. Even with more stringent thresholds (e.g., coding divergence minus 1 SD or half of coding divergence) between the reference genomes and the most diverged species, although the differences become smaller, the patterns remain essentially the same (see supplementary fig. S2, Supplementary Material online). However, the differences between the numbers of primate and carnivore CNSs become smaller.

The differences in the abundance may be due to the difference in genome data quality. We relied on the genome data coverage information available in the database. All genomes have more than 6× coverage. Although human has the best genome quality, it is important to note that CNSs are conserved in all members of a lineage, and therefore, the genome qualities of all members contribute to the number of CNSs. Also, though mouse and rat have very good genome qualities, rodents have the least number of CNSs. We further checked the effect of genome quality by searching for the number of CNSs conserved with chicken in all the species. In all the lineages, the number of elements conserved between each species and chicken is similar (data not shown). Human and dog have similar number of CNSs conserved with chicken (see supplementary table S4, Supplementary Material online) but the number of primate common CNSs is higher than that of carnivores. These suggest that genome quality and repeat masking database do not significantly influence the results.

We next checked the distribution of CNSs by length. We compared the abundance of CNSs at various length categories using the whole coding threshold. Our result shows that primates have the highest number whereas rodents have the least, irrespective of the length criterion used (see supplementary fig. S3, Supplementary Material online). The average lengths of CNSs are 195, 219, 228, and 204 bp for primates, rodents, carnivores, and cetartiodactyls, respectively, though the SDs are high. This result shows that although primates have higher proportion of short CNSs, irrespective of the length threshold used, primates have more CNSs and rodents have the least. Therefore, choosing shorter length threshold would not alter the pattern of our result.

### Phylogenetic Origin and Abundance of CNSs

**Fig. 2.—:**
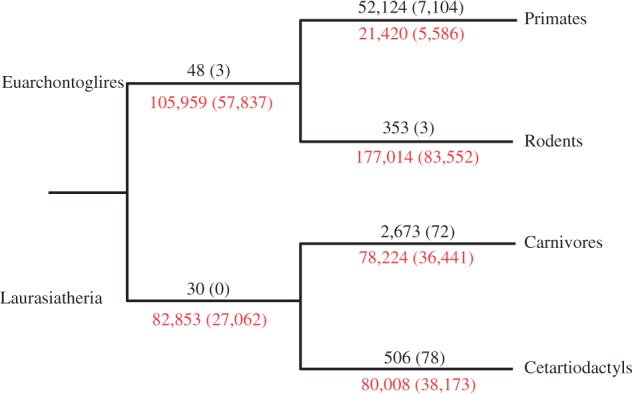
The phylogenetic gain and loss of CNSs. The values on a branch (in black font) are the numbers of CNSs gained on the branch, whereas the values under a branch (in red font) are the number of CNSs lost on that branch with African elephant as the reference. For each point, the values are the numbers if whole coding thresholds were used, whereas the values in parentheses are the numbers if skip3 thresholds were used.

The difference in the abundance of CNSs obtained across lineages may be due to the difference in the amount of CNSs that were gained or lost at each branch. We therefore checked the CNSs that are unique to each lineage. These are sequences that are reasoned to have gained new functional constraint on that branch and all the extant members still retain the function. With the whole coding threshold, the amount of primate-unique CNSs (52,124) is more than 100-fold that of rodents (fig. 2). Among 861,183 primate-common CNSs, only 52,124 were found to be primate-unique and only 1,779 are shared by all the species used. Of the total 37,709 eutherian common CNSs, only 12,378 evolved in eutherian common ancestor. This dynamics indicates that many CNSs are of older origin but have been lost in some species, making it difficult to trace their evolutionary origin. If we consider the number of shared CNSs, the more diverged the species included, the less the number of CNSs that can be retrieved. Although primates have the highest number of shared CNSs, 73,641 euarchontoglire (primates, rodents, and rabbit) common CNSs are lower than 111,705 laurasiatheria (carnivores, cetartiodactls, and microbat) common. The number of CNSs conserved in all eutherian species (euarchontoglire, laurasiatheria, and African elephant) is 37,709. When we included opossum and platypus, the number of CNSs became 11,693. These patterns are similar when the skip3 threshold was used. This is expected due to evolution of new CNSs as well as high turnover rate of CNSs.

What might be responsible for the high number of CNSs in primate lineage? One possibility is that there were many duplications of the CNSs in the primate common ancestor. To check this, we searched for duplicates in primate unique CNSs. Out of 52,124 primate unique CNSs retrieved using the whole coding threshold, 49,888 (96%) are single copies with no duplicates. We then checked how old the primate unique CNSs are. We did not include mouse lemur, a primate which diverged earlier from human, in our analysis due to lower genome quality and divergence time consideration of its genome. We therefore checked how many of the primate unique CNSs have copies in its genome. We found 18,308 CNSs with hits in the mouse lemur genome, out of which 9,528 are 100 bp or longer. This value is much higher than the unique CNSs in other lineages, suggesting that the observed higher number of CNSs in primates is not only solely because of the divergence time but also because of functional constraints. On the other hand, most of the CNSs evolved after the emergence of mouse lemur are suggested to acquire new functional constraints.

### Lineage-Specific Loss of CNSs

The second contributing factor to the difference in number of CNSs in each lineage may be the retention or loss of ancestral CNSs. The loss of ancestral CNSs could happen through two processes. The first process is through high-sequence divergence. The constraint on a sequence might be relaxed in a lineage from loss of function or adaptive evolution. In this case, although the homologous sequence is retained in the genome, the sequence has diverged beyond recognition. The second process that may lead to CNS loss is sequence deletion as reported by Hiller et al. (2012). In this case, the whole region or a part of the region might be deleted from the genome. By lowering the threshold, we could retrieve some of the CNSs that have been lost by sequence divergence. However, not being able to retrieve lost CNSs, even after lowering the threshold, may not necessarily mean they were lost by deletion. Some CNSs might have gone through high-sequence divergence such that sequence similarity would have been completely lost or lost to the level that it cannot be identified by homology search.

In our definition of lost CNSs, the specific mechanism of loss is not considered. A CNS may thus have been lost through high-sequence divergence or deletion. We assume that, in either case, the function would have been modified if not completely lost. We therefore checked the rate of loss of amniote ancestral CNSs conserved between chicken and at least one other mammalian species. For this analysis, we used eight species, including a representative species from each of the four lineages (see fig. 2; supplementary table S4, Supplementary Material online; Material and Methods). As chicken and other species have lost some CNSs, complete set of amniote ancestral CNSs cannot be accounted for. However, assuming an unbiased parallel loss between chicken and the mammalian species used, this analysis would give a true pattern of lineage-specific rate of loss. Chicken has 66,210 CNSs, conserved in one or more of the species used and 11,472 conserved in all, implying that 54,738 CNSs have been lost in one or more species or lineages. We then calculated the number of CNSs lost in each branch. Mouse has lost more number of CNSs than any other species (table 1 and supplementary table S4, Supplementary Material online). In euarchontoglire common ancestor, 7,699 CNSs were lost, whereas 3,833 CNSs were lost after the divergence of euarchontoglire and laurasiatheria and before the divergence of cow and dog. However, 1,861 CNSs lost in the human lineage after the divergence from mouse is lower, and the 13,015 CNSs lost in the mouse lineage is higher than those lost in cow and dog lineages after their divergence. Similar patterns are observed with the skip3 threshold (table 1 and supplementary table S4, Supplementary Material online). Out of the total 41,465 CNSs in chicken, only 7,889 are conserved in all. This suggests that the rates of CNS loss are heterogeneous in euarchontoglire lineages (see supplementary fig. S1, Supplementary Material online), with human lineage having a lower rate and mouse lineage having a higher rate.

**Table 1:** The Loss of Ancestral CNSs with Different Thresholds and Reference Species

	Number of CNSs Lost			
	Chicken as Reference		African Elephant as Reference	
Phylogenetic Branch	Whole Coding	Skip3	Whole Coding	Skip3
Primates	1,861	1,243	21,420	5,586
Rodents	13,015	7,240	177,014	83,552
Carnivores	4,806	3,300	78,224	36,441
Cetartiodactyls	6,884	3,882	80,008	38,173
Euarchontoglires	7,699	5,236	105,959	57,837
Laurasiatheria	3,833	1,786	82,853	27,062
Euarchontoglires and Laurasia	1,888	1,476	—	—
African elephant	12,605	7,938	—	—
Eutheria	7,468	5,171	—	—
Opossum	21,348	13,142	—	—
Theria	7,437	3,798	—	—
Platypus	30,514	18,698	—	—

We then used African elephant as the outgroup genome so as to include more recent CNSs in our focus. The results in figure 2, table 1 and supplementary table S4, Supplementary Material online, indicate a very rapid loss of CNSs in a species-specific manner. With the whole coding threshold, out of 439,034 CNSs present before the eutherian common ancestor, only 109,475 (∼25%) was retained in all the four species. A significant proportion of the CNSs (36.4%) have been lost in three of the four species; 19.3% have been lost in two species; and 19.3% have been lost in just one species. When the skip3 threshold was used, of the 195,926 CNSs in the eutherian common ancestor, 35%, 21%, 22%, and 22% are found in one, two, three, and all of the four species, respectively. This suggests rapid independent loss events of CNSs. Even when the number of CNSs per unit branch length was considered, the patterns did not change. This suggests that real functions, in addition to evolutionary rate differences, account for the dynamics of CNSs.

### CNSs Are Under Functional Constraint

**Fig. 3.—:**
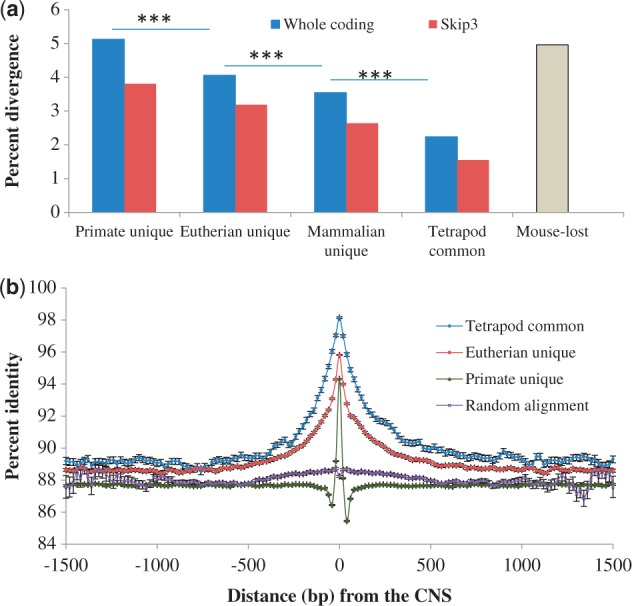
The conservation levels in and around CNSs. (*a*) The divergence levels of CNSs (***t-test *P*-value < 10^−20^). (*b*) The conservation levels of flanking regions of CNSs with whole coding thresholds. Point 0 is the average percent identity of 100 bp at the center of the CNSs, whereas other points are the average of 50-bp windows moved at 20-bp steps starting from 30 pb inside the CNSs. The bars are the standard error of the mean for each window.

We first investigated whether the CNSs of different ages are under similar functional constraint. We compared the conservation level of CNSs that evolved in primate-, eutherian-, mammalian-common ancestor, and those that were found in tetrapod common ancestor. The numbers of CNSs retrieved using whole coding thresholds were 52,124, 12,378, 4,059, and 1,779, whereas the numbers retrieved using skip3 thresholds were 7,104, 2,118, 1,390, and 1,733, for primate unique, eutherian unique, mammalian unique, and tetrapod common, respectively. Our choice of primate order was because of higher number of primate unique CNSs. We extracted human and marmoset sequences for each of the classes and calculated the pairwise divergence using ClustalW (Larkin et al. 2007). As shown in figure 3*a*, percent difference of tetrapod common CNSs is the lowest while that of primate unique CNSs is the highest (*t*-test *P* < 10^−^^20^). This suggests that more ancestral CNSs are under stronger constraint than the recent ones.

**Fig. 4.—:**
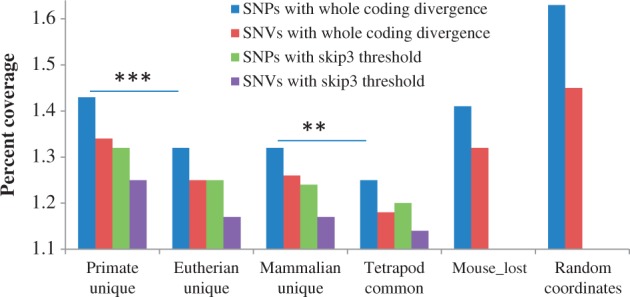
The SNP coverage of CNS. The average numbers of SNPs found in 100 bp of CNS are presented for each age category. Complete SNP data as well as SNV data were used. Random coordinates of number and lengths similar to whole coding primate unique CNSs were used (***t-test *P*-value < 0.001; **t-test *P*-value < 0.005).

Is the difference in conservation level due to age alone or as a result of indispensability of the function? To answer this, we compare the conservation level of CNSs that have been lost in mouse but conserved among human, marmoset, and chicken (see table 1). The mouse-lost CNSs are not significantly different from primate unique CNSs, even though primate unique CNSs are more recent. This suggests that the difference in conservation levels is due to the indispensability of the function. The lineage-specific evolution and loss of CNSs are major players in shaping the abundance of CNSs in a lineage (fig. 2; table 1). Because recently evolved CNSs and those that have been lost in some lineages are under relatively lower constraint (figs. 3 and 4*a*), too stringent threshold would imply that most of the CNSs retrieved would be ancestral ones, and therefore lineage difference will be reduced. Indeed, that is the observation, especially between primates and carnivores, when we used more stringent thresholds (coding divergence minus 1 SD or half of coding divergence; see supplementary fig. S2, Supplementary Material online).

As primate unique CNSs are under lowest functional constraint, we checked whether the conservation level is different from that of randomly retrieved sequences. We first randomly retrieved noncoding sequences of the same number and lengths as the primate unique CNSs retrieved using the whole coding threshold. We did homology search and used sequences with unique identifiable homolog. Only 16,934 (34%) have unique identifiable homolog of at least 100 bp long. The average percent difference of randomly retrieved sequences (12%) is significantly higher than 5.8% of primate unique CNSs (*t*-test *P* = 0).

We also checked the conservation level of the CNS flanking regions. Figure 3*b* (for the whole coding threshold) and supplementary figure S4, Supplementary Material online (for the skip3 threshold) show that CNSs are under stronger conservation level, compared with the flanking regions. Also, the conservation of the flanking regions of tetrapod common CNSs is higher than that of the primate unique CNSs as predicted from figure 3*a*. Although more ancestral CNSs are peaks of long conserved regions, younger CNSs seem to be peak of poorly conserved regions. These patterns are not observed in random sequences. Note that for random sequences, we used unfiltered alignments of at least 1,200 bp long. Because the sequences have the tendency of containing CNSs, there seems to be slight elevation around the CNSs. Even with that, the center is not the peak.

As another measure of functional constraint, we examined the coverage of SNPs and SNVs as found in an Ensembl database of SNP. Primate unique CNSs had higher percentage of SNPs and SNVs, and tetrapod common CNSs have lower SNPs and SNVs as expected (fig. 4). Again, mouse-lost CNSs are similar to primate unique CNSs supporting the hypothesis that the indispensability of the CNS, rather than just the age, determines the strength of functional constraint. It is also important to note that CNSs cover less SNPs and SNVs, compared with random sequences (*t*-test *P* < 10^−^^15^).

Another test of functionality of a sequence is DAF analysis (Drake et al. 2006; Takahashi and Saitou 2012). The frequency of derived alleles of functional regions is expected to be lower than the genomic average because of purifying selection. Our result (see supplementary fig. S5, Supplementary Material online) supports that those CNSs are under purifying selection. Higher proportion of CNSs has lower derived alleles than random expectation. For example, for alleles with frequency of <0.1, the number of alleles with frequency of <0.1 in CNSs is significantly higher than random expectation (binomial *P* < 10^−^^15^). On the other hand, at higher frequencies, CNSs have slightly lower proportion than the genomic average.

**Fig. 5.—:**
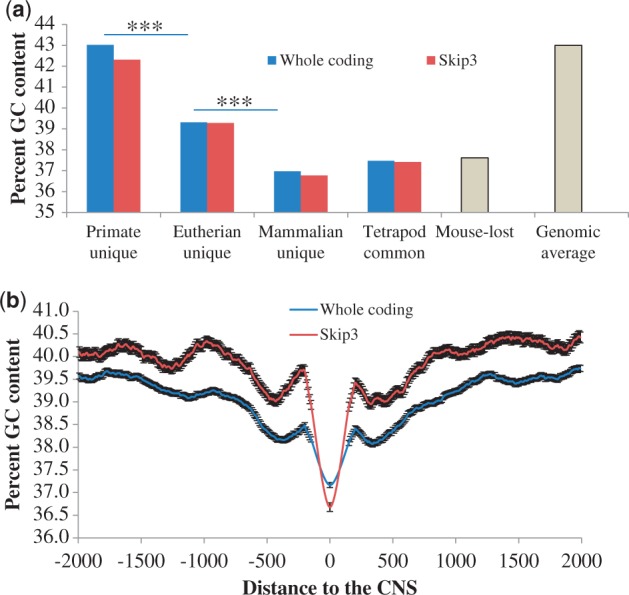
GC contents of CNS and flanking regions. (*a*) The GC contents of CNSs. The genomic average is from Karro et al. (2008) (***t-test *P*-value < 0.001). (*b*) Using sliding windows of 200-bp size and sliding steps of 10 bp, the percent GC contents of the mammalian unique CNSs and flanking regions were computed. Position 0 is the 100 bp in the center of the CNSs and the first window starts from 50 bp into the CNSs.

We checked the nucleotide composition of the CNSs, with emphasis on GC content. The GC content of the CNSs is significantly lower than that of protein-coding regions (*t*-test *P* < 0.001). Except for the primate unique CNSs, GC contents of CNSs unique to the other three mammalian orders are lower than that of genomic averages reported by Karro et al. (2008). This might be because primate unique CNSs evolved recently and did not have enough time to lower their GC contents. As in the conservation level and SNV coverage, the GC content tends to correlate with the age of the CNSs (fig. 5*a*). This result suggests that the GC content of CNSs decays as CNSs become old. In fact, supplementary table S5, Supplementary Material online, shows that GC→AT substitutions are more than AT→GC substitutions.

In addition, we conducted sliding window analysis to examine the GC content of the flanking regions of the CNSs. We focused on mammalian unique CNSs with the lowest GC content (fig. 5). With the sliding windows of size 200 bp, 10 bp step size starting from 50 bp inside the CNSs, we found a sharp decrease toward the CNSs (fig. 5*b*). Similar pattern was observed when we considered all skip3 primate common CNSs (see supplementary fig. S6, Supplementary Material online). The observed decrease in the GC contents toward CNSs might be related to nucleosome occupancy as reported by Gupta et al. (2008).

### Genomic Distribution

**Fig. 6.—:**
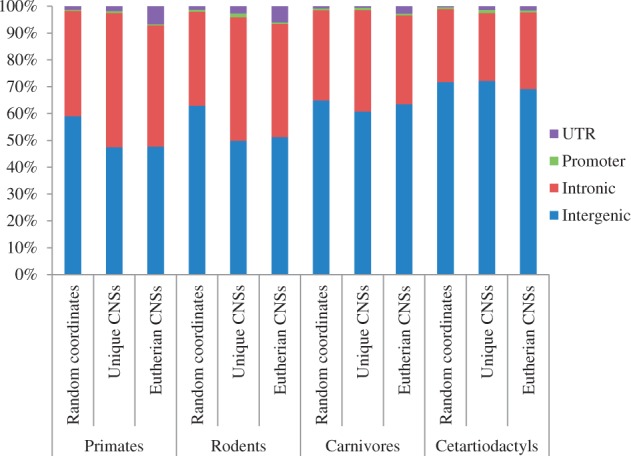
The genomic distribution of the CNSs. Using whole coding thresholds, percentage of CNSs found in each genomic region for each lineage and category are presented. Eutherian CNSs are the 35,906 single-copy CNSs conserved in all the four lineages. For random coordinates, same number and lengths of eutherian CNSs were randomly selected from noncoding sequences of each reference species.

The genomic distribution of the CNSs varies across lineages. This is especially obvious when considering CNSs found in intergenic and intronic regions in each lineage (fig. 6 and supplementary fig. S7, Supplementary Material online). Primates and rodents (euarchontoglires) have higher proportion of intronic CNSs than random expectation (binomial *P* < 10^−^^100^). On the contrary, carnivores and cetartiodactyls (laurasiatheria) do not have such a high proportion of intronic CNSs. The distribution of carnivore and cetartiodactyl CNSs is very close to the random expectation, which might be due to the quality of genome data, especially of cow. Primates also have notably higher promoter unique CNSs. There is a difference between the genomic distribution of recently evolved CNSs and older ones, especially when we focus on UTR CNSs. The UTR proportion of the eutherian common CNSs in primates and rodents agrees well with Siepel et al. (2005), who reported that vertebrate highly conserved elements were associated with 3′ UTR of regulatory genes. Our analysis suggests that the locations of functional elements are dynamic even among eutherian mammals.

From figure 6, it can be observed that there is a difference between proportions of UTR CNSs in unique and ancestral CNSs. For eutherian common CNSs in primates, for example, 7% of the CNSs are located in the UTRs, compared with 2% of the unique CNSs located in the same location (binomial *P* < 10^−^^100^). The same pattern was observed across the four orders. This suggests that more ancestral CNSs are more associated with UTRs. Apart from age-related dynamics in genomic distribution of CNSs, we also observed species-related dynamics (fig. 6).

To examine whether ancestral CNSs are always fixed in location or may be “relocated,” in terms of location with respect to genes, we considered the genomic location of eutherian common CNSs, using CNSs with single copy, such that CNSs with duplicates in any of the species representing each lineage are not included. It turned out that the location of orthologous CNSs is not always fixed (fig. 5 and supplementary fig. S8, Supplementary Material online; table 2 and supplementary tables S6 and S7, Supplementary Material online). Out of 35,906 homologous single-copy CNSs considered, 10,701 (about 30%) are located in a different region in one or more species. Taking human and dog, for example, 19.38% of homologous CNSs are not located in the same genomic location in the two species (table 2). The events of difference in locations between mouse and human genomes are not as many. We acknowledge the fact that this pattern may be caused by genome annotation quality heterogeneity. Human and mouse genomes have been reasonably well annotated, and dog annotation has been recently improved with cDNA data. Even between human and mouse, 8% of the CNSs are located on different locations.

**Table 2:** The Similarity of the Genomic Location of Orthologous CNSs

	Human	Mouse	Dog	Cow
**Human**		2,883 (8.03%)	6,960 (19.38%)	8,387 (23.36%)
**Mouse**	*33,023*		5,951 (16.57%)	7,221 (20.11%)
**Dog**	*28,946*	*29,955*		5,099 (14.20%)
**Cow**	*27,519*	*28,685*	*30,807*	

Note.—The genomic locations of the 35,906 single-copy CNSs that are shared by all eutherian species used were compared in the four representative species. The numbers in italics are located on the same genomic location, whereas the numbers in the upper part (roman) are located on different locations.

We further examined whether the CNSs are uniformly distributed over the chromosome by using 1-Mbp windows with 100 kbp step size. CNSs and genes are distributed nonuniformly across the chromosomes but not found at all around centromeres (see supplementary fig. S9, Supplementary Material online). This might be because centromeres have many repeat elements. Some conserved regulatory elements have been reported to be found in the gene desert (Ovcharenko et al. 2005), and Siepel et al (2005) reported that vertebrate highly conserved elements are associated with stable gene desert. We therefore examined the correlation between the number of CNSs and the genes in 1-Mbp bin with at least a gene and a CNS. The Pearson correlation coefficients (and *P* values) are −0.2234 (8.069 × 10^−^^299^), −0.2425 (2.18 × 10^−^^300^), −0.3737 (0.00), and −0.2486 (0.00) for primates, rodents, carnivores, and cetartiodactyls, respectively. The negative correlations in all the lineages indicate that more CNSs cluster where there are few genes (gene deserts). However, considering the proximity of CNSs to genes, more than 90% of group-common and lineage-specific CNSs have the closest gene within 400-kbp upstream or downstream in all lineages except in cetartiodactyls. In fact, more than 95% of CNSs have the closest gene within 700 kbp (see supplementary fig. S10, Supplementary Material online).

### Functional Analysis of the CNSs

Takahashi and Saitou (2012) previously reported lineage-specific highly conserved noncoding sequences (HCNSs), which were associated with some protein-coding genes. As the same primate species were used in both studies, we checked the overlap of the primate sequences found in both studies. Of the total 8,198 HCNSs reported, 6,643 (81%) were retrieved in this study. Among nonoverlapping 1,555 (19%), 1,005 were nonprotein-coding genes that were removed from the present analysis.

To check whether identified CNSs have regulatory functions, we checked for the overlap with reported human ChIP sequences from UCSC table and found 166,451 primate common CNSs overlap with previously reported ChIP-seq data. As another signature of regulatory activity, we checked for the overlap with DNAse clustered sites and found 317,590 overlaps (see supplementary fig. S11, Supplementary Material online). We also checked for the possibility of transcription of the identified CNSs using the GENCODE comprehensive gene data from UCSC table. Only 51,261 of the CNSs retrieved with the whole coding threshold were found to overlap any ENCODE gene. It is important to note that GENCODE genes we used cover about 48% human genome while the ChIP-seq data cover 7.78%. These results suggest that identified CNSs are 20 times more likely to be regulatory elements, compared with genes. In fact, among the 51,261 CNSs found to overlap ENCODE genes, 16,278 also overlap ChIP-seq data. If we checked for more regulatory signatures such as histone modification marks, DNA-hypersensitive sites, or focus on more cell or tissue types as well as more developmental stages, we expect that many more CNSs may have regulatory signatures. Thus, many of the identified CNSs most likely have regulatory functions. Supplementary figure S12*a*, Supplementary Material online, shows an example of a multiple alignment of an identified CNS. In addition, Supplementary figure S12*b* and *c*, Supplementary Material online, shows examples of identified CNSs that overlap some regulatory signatures and experimentally confirmed brain enhancers, as reported by Viesel et al (2013).

To further investigate the likely functions of the CNSs, we studied the enrichment of the biological process of the genes that are likely regulated by the CNSs using PANTHER. Several studies have reported that CNSs are associated with genes involved in transcription regulation (e.g., Vavouri et al. 2007; Elgar 2009) and developmental genes (Hardison 2000; Levy et al. 2001). We studied the enrichment of the likely target genes of tetrapod common CNSs using PANTHER and considered the biological process of the 20 most enriched ontology groups. The gene ontology result shows that the ancestral CNSs are enriched in genes involved with transcription regulation and development (table 3), suggesting that the CNSs play an important role in phenotypic diversity. It is important to note here that nervous system related genes are also enriched, suggesting that many regulatory elements associated with nervous system are conserved. This observation is consistent with the report of Matsunami and Saitou (2013) that vertebrate paralogous CNSs may be related to gene expression in the brain. PANTHER database uses a limited number of genes. Moreover, we analyzed the enrichment by CNS-weighted genes. We therefore calculated the enrichment and *P* value under binomial distribution as described by PANTHER for the top three overrepresented terms and top two underrepresented terms (see supplementary table S8, Supplementary Material online). The three overrepresented terms are transcription, development, and nervous system, whereas the two underrepresented terms are response to stimulus and immune and defense. This observation suggests that CNSs tend to be associated with genes that are under negative selection and underrepresented in genes under positive selection.

**Table 3:** Gene Ontology of Tetrapod Common CNS-Associated Genes

Biological Process	*P* Value	Enrichment
Regulation of transcription from RNA polymerase II promoter	1.97e−27	2.67
Transcription from RNA polymerase II promoter	2.81e−24	2.30
Nervous system development	2.85e−24	2.75
Transcription	4.56e−24	2.29
Ectoderm development	4.72e−24	2.60
System development	4.11e−22	2.26
Developmental process	2.21e−20	1.96
Muscle organ development	1.72e−12	3.30
Embryonic development	4.27e−12	3.32
Nucleobase, nucleoside, nucleotide, and nucleic acid metabolic process	9.46e−12	1.62
Mesoderm development	2.00e−11	2.10
Segment specification	7.70e−09	4.22
Pattern specification process	3.62e−08	2.93
Heart development	2.08e−06	3.33
Primary metabolic process	2.13e−06	1.27
Metabolic process	9.87e−06	1.25
Gut mesoderm development	2.66e−05	4.87
Protein modification process	5.94e−04	1.72
Neurological system process	7.13e−04	1.54
Visual perception	1.21e−03	2.21

Note.—*P* value was calculated using binomial statistics, whereas the enrichment is the ration of actual number of gene to the number of genes expected under random expectation.

## Discussion

Understanding the molecular mechanism underlying the phenotypic diversity observed among species has been of interest to many scientists. Before the invention of high technology that is now available for molecular studies, the taxonomists have classified the organisms according to the phenotypes of each species, classifying species with similar features into the same group. As phenotypes have genetic background, it is expected that each taxonomic group should share some unique genetic features. In fact, many phylogenetic studies have been in agreement with taxonomic classification (e.g., Meyer and Zardoya 2003; Blanga-Kanfi et al. 2009). Therefore, the knowledge of some unique molecular features should shed more light into the understanding of lineage evolution. However, the phenotypic diversity observed among species and taxonomic groups could not be sufficiently explained by mere presence or absence of a particular set of genes (Stern 2000; Wray 2003). This is because many genes are highly conserved in many species. The regulation of spatiotemporal gene expression has long been suggested to be important in phenotypic diversity (Zuckerkandl and Pauling 1965; King and Wilson 1975). Therefore, one way to molecularly explain the phenotypic diversity may be in terms of gene regulation. Not just the presence or absence of a gene but also when, how, and where certain genes are expressed are also important in the evolution of phenotypic diversity observed among and within orders (Zhang and Peterson 2006; Takahashi and Saitou 2012; Wittkopp and Kalay 2012). A genetic theory of morphological evolution states that form evolves largely by mutations in cis*-*regulatory sequences that alter the expression of functionally conserved proteins (Carroll 2008). In fact, Cretekos et al. (2008) reported that regulatory divergence modifies limb length between mammals. CNSs are therefore good proxy for conserved regulatory elements.

In this study, we defined CNSs as homologous regions with at least 100 bp length and average conservation level of the protein-coding genes with one-to-one correspondence. Divergence thresholds were set by using whole coding genes as well as third codon-skipped coding sequences. Although it is possible that some functional CNSs may have less degree of conservation, we assume that only CNSs with threshold or lower divergence levels have a function that is important for the group. We therefore do not discard the possibility of functional sequences with conservation lower than the threshold, as it is known that many regulatory elements are not evolutionarily conserved (Schmidt et al. 2010; Shen et al. 2012). The percent identity threshold minimizes the false negatives and also allows for correction for difference in evolutionary rates among lineages. The conservation level, coverage of SNV, GC content, and overlap with previously reported regulatory signatures as well as DAF analysis support that the CNSs identified are under functional constraint and may have regulatory functions.

To check the orthology of the CNSs, we constructed the phylogenetic tree using concatenated CNSs. If the CNSs are orthologous, we would expect that the species tree should be recapitulated with high statistical support. Indeed, all branches of the tree (see supplementary fig. S13, Supplementary Material online) had 100% bootstrap probabilities, and it had the identical topology with the established mammalian phylogeny (e.g., Beck et al. 2006). This result is consistent with the hypothesis that the CNSs of different species are orthologous. In addition, the tree clearly shows that CNSs can be useful for producing species trees.

Although there are several reports on CNS evolution in vertebrates and tetrapods, our study shows a significant difference in the evolutionary dynamics of CNSs among the four mammalian lineages that diverged <120 Ma. There is an obvious difference in the abundance of CNSs in each lineage. Although primates have more CNSs, rodents have the least CNSs. Also, the rate of gain of function of noncoding regions, detectable as regions uniquely conserved in a lineage, varies across lineages with primates having much more than any other lineages. Takahashi and Saitou (2012) also found that the numbers of HCNSs are different with rodents having more than primates. It is important to note that although Takahashi and Saitou (2012) used MEGABLAST for homology search, we used BlastN, which is more sensitive but much slower. Apart from the difference in methodology, especially the definitions of HCNSs (Takahashi and Saitou 2012) versus CNSs (this study), the different pattern may be due to the species included especially in the rodent lineage. We included guinea pig with higher genetic distance to mouse. As we have shown that species-specific loss of CNSs occurs, the number of species included would affect the abundance of CNSs that could be recovered. The loss of ancestral CNSs was higher in mouse than in human. Our study also showed that more CNSs originated in the common ancestor of primates compared with that of rodents. These differences may contribute to the lineage-specific phenotypic dynamics.

There are still many uncertain areas in the evolution and actual function of CNSs. Although there are many functional signatures on the sequences, some experiments involving deletions of such conserved elements yielded viable mice (Nóbrega et al. 2004; Ahituv et al. 2007). These reports raised some concerns about the functions of CNSs and the actual reasons behind their conservations. However, the fact that the deletion of the regions did not produce any phenotype does not necessarily imply that they are functionless. Their functions might be subtle and related to a weak fitness in the wild. As a matter of fact, deletion of some enhancer elements of *Drosophila shavenbaby* genes produced no phenotype under optimal temperatures but under low or high temperatures, non-wild-type phenotypes were produced (Frankel et al. 2010). Although it is possible that some CNSs may have other functions, they are more likely to function as regulatory elements (Hardison 2000; Levy et al. 2001; Hemberg et al. 2012). That the CNS clustering around genes involved in transcription regulation and development implies that they may contribute to phenotypic differences. There is a possibility that some of the identified CNSs might be some genes that are yet to be annotated. However, the overlap of the CNSs with reported regulatory signatures supports that many have gene regulatory functions. Attributing more CNSs to more regulatory elements, our study suggests that primates have more shared regulatory elements implying higher complexity in primates compared with other lineages. Although the human genome has about the same number of protein-coding genes as the mouse genome, the higher number of human regulatory elements (inferred from the higher number of CNSs) suggests that primates have complex shared gene regulatory system that deals with nervous system and brain development. This is reasonable because we have shown CNSs to be enriched in genes associated with the nervous system as previously reported.

Schmidt et al. (2010) reported 11,588 CEBPA bindings in human cells and 19,212 transcription factor bindings for the same protein in mouse cells using ChIP-seq analysis in livers. This suggests that, for some proteins, human may not necessarily have higher transcription factor binding sites. This means that fewer CNSs do not always imply fewer regulatory elements. It may just show fewer shared regulatory elements as a result of high turnover rates. This makes more sense with our discovery that more ancestral CNSs are under stronger constraint because of their indispensability and that newly gained sites have higher turnover rates. Because functional elements have higher turnover rates (Meader et al. 2010), we interpret fewer CNSs in rodents, not as an effect of just fewer regulatory elements but as a result of high turnover rates. This implies that many regulatory elements are not conserved in rodents. This result suggests a high morphological diversity in the rodent lineage.

The genomic location may give a hint about the function of a CNS. For example, CNSs located several hundred kbp to a gene is less likely to regulate that gene as a proximal promoter element. Our study indicates that the genomic distribution of CNSs is different in each lineage. Phylogenetically close lineages, such as primates and rodents, have more similar distribution. Also, about 30% of single-copy orthologous CNSs are located on different genomic positions in one or more different species. Although lower quality of cow genome annotation may contribute to this difference, even between human and mouse that have been well studied, 8% orthologous CNSs are located on different locations. This suggests that in the course of evolution, the genomic location of CNSs with respect to genes might change. One mechanism through which such “relocation” can happen is through gene loss. The gene harboring a CNS might be lost and the CNS kept if the CNS regulates a neighboring active gene. In this way, CNS previously in an intron is “relocated” to an intergenic region. Other gene restructuring processes such as translocation, inversion, gene fusion, and duplication followed by loss might also lead to this relocation. It is possible that such relocation may be correlated with local evolutionary rates as Liu et al. (2006) reported difference in evolutionary rates among regions. This assertion has to be further investigated.

Our gene ontology analysis indicates that the putative target genes of the ancestral CNSs are enriched in genes involved with transcription and development. As the phenotypic differences observed among species arise during development, the CNSs may contribute to phenotypic differences. Hiller et al. (2012), for example, showed that the CNEs function as a spinal cord enhancer. Although CNSs may have other functions apart from regulation, they are more likely to function as regulatory elements (Hemberg et al. 2012; Shen et al. 2012). Considering the high dynamics in abundance, retention, and loss of ancestral CNSs and gain of new ones as well as the difference in genomic distribution, many CNSs are expected to hold the key to the understanding of the phenotypic diversity observed among species, via their activities as regulatory elements or other mechanisms that are yet to be fully understood.

## Supplementary Material

Supplementary figures S1–S13 and tables S1–S8 are available at *Genome Biology and Evolution* online (http://www.gbe.oxfordjournals.org/).

Supplementary Data
