# Supplementary material for: Heterogeneous Tempo and Mode of Conserved Noncoding Sequence Evolution among Four Mammalian Orders
Source: Genome Biol Evol. 2013 Nov 20;5(12):2330–43. doi: 10.1093/gbe/evt177 (PMC3879966; doi:10.1093/gbe/evt177)
Supplement: Supplementary Data [file supp_5_12_2330__index.html]

Heterogeneous Tempo and Mode of Conserved Noncoding Sequence Evolution among Four Mammalian Orders — Supplementary Data 

# Heterogeneous Tempo and Mode of Conserved Noncoding Sequence Evolution among Four Mammalian Orders

## Supplementary Data

files

**Files in this Data Supplement:**

- Supplementary Data - pdf file
