# Supplementary material for: Heterogeneous Tempo and Mode of Conserved Noncoding Sequence Evolution among Four Mammalian Orders
Source: Genome Biol Evol. 2013 Nov 20;5(12):2330–43. doi: 10.1093/gbe/evt177 (PMC3879966; doi:10.1093/gbe/evt177)
Supplement: Supplementary Data [file supp_evt177_Babarinde_Supplementary_no_mark.pdf]

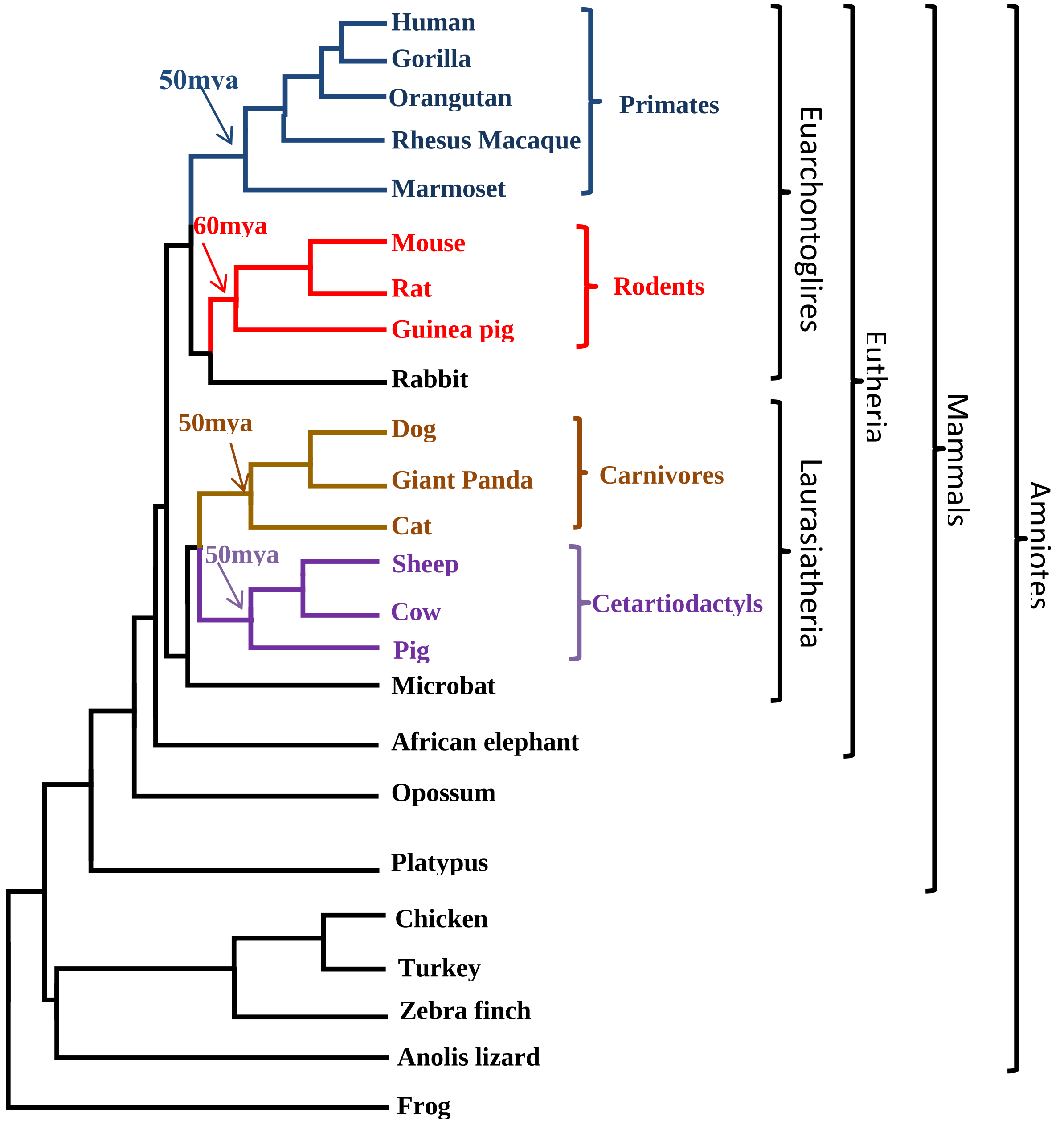

Figure S1: The phylogenetic and taxonomic classification of the species used in this study. The most diverged species in each of the four mammalian lineages used in this study is around 50~60mya. The tree is not scaled.

Table S1: Genome sequences used for the analyses

Species	Database	Build	Order
Human	*Ensembl*	Homo_sapiens.GRCh37.66.dna_rm.toplevel.fa	Primates
Gorilla	*Ensembl*	Gorilla_gorilla.gorGor3.1.66.dna_rm.toplevel.fa	Primates
Orangutan	*Ensembl*	Pongo_abelii.PPYG2.66.dna_rm.toplevel.fa	Primates
Rhesus macaque	*Ensembl*	Macaca_mulatta.MMUL_1.66.dna_rm.toplevel.fa	Primates
Marmoset	*Ensembl*	Callithrix_jacchus.C_jacchus3.2.1.66.dna_rm.toplevel.fa	Primates
Mouse	*Ensembl*	Mus_musculus.NCBIM37.66.dna_rm.toplevel.fa	Rodentia
Rat	*Ensembl*	Rattus_norvegicus.RGSC3.4.66.dna_rm.toplevel.fa	Rodentia
Guinea pig	*Ensembl*	Cavia_porcellus.cavPor3.66.dna_rm.toplevel.fa	Rodentia
Rabbit	*Ensembl*	Oryctolagus_cuniculus.oryCun2.66.dna_rm.toplevel.fa.	Lagomorpha
Dog	*Ensembl*	Canis_familiaris.CanFam3.1.69.dna_rm.toplevel.fa	Carnivora
Panda	*Ensembl*	Ailuropoda_melanoleuca.ailMel1.70.dna_rm.toplevel.fa	Carnivora
Cat	*Ensembl*	Felis_catus.Felis_catus_6.2.70.dna_rm.toplevel.fa	Carnivora
Cow	*Ensembl*	Bos_taurus.UMD3.1.66.dna_rm.toplevel.fa.	Cetartiodactyla
Sheep	UCSC genome	oviAri1.fa.masked	Cetartiodactyla
Pig	*Ensembl*	Sus_scrofa.Sscrofa9.66.dna_rm.toplevel.fa	Cetartiodactyla
Microbat	*Ensembl*	Myotis_lucifugus.Myoluc2.0.66.dna_rm.toplevel.fa	Chiroptera
African elephant	*Ensembl*	Loxodonta_africana.loxAfr3.66.dna_rm.toplevel.fa	Proboscidea
Opossum	*Ensembl*	Monodelphis_domestica.BROADO5.66.dna_rm.toplevel.fa	Didelphimorpha
Platypus	*Ensembl*	Ornithorhynchus_anatinus.OANA5.66.dna_rm.toplevel.fa	Monotremata
Chicken	*Ensembl*	Gallus_gallus.WASHUC2.66.dna_rm.toplevel.fa	Galliformes
Turkey	*Ensembl*	Meleagris_gallopavo.UMD2.66.dna_rm.toplevel.fa	Galliformes
Zebra finch	*Ensembl*	Taeniopygia_guttata.taeGut3.2.4.66.dna_rm.toplevel.fa	Passeriformes
Anolis lizard	*Ensembl*	Anolis_carolinensis.AnoCar2.0.66.dna_rm.toplevel.fa	Squamata
Frog	*Ensembl*	Xenopus_tropicalis.JGI_4.2.66.dna_rm.toplevel.fa	Anura

Table S2: Setting of whole coding divergence threshold for each lineage

	Synonymous (S)	Nonsynonymous (N)	Genomic noncoding	Whole coding divergence (C)	Mean divergence proportion (P)
Primates	0.2224 (1.81)	0.0441 (0.20)	0.1218	0.0575 (0.03)	0.0603
Carnivores	0.3868 (2.06)	0.0572 (0.15)	0.1861	0.0817 (0.04)	0.0633
Cetartiodactyls	0.5201 (2.87)	0.0687 (0.24)	0.2143	0.0979 (0.05)	0.0561
Rodents	0.9601 (2.99)	0.1101 (0.25)	0.239	0.1656 (0.06)	0.0578

The mean values of the divergences are given while the values in parentheses are the standard deviations. Synonymous substitution, genomic noncoding divergence and coding divergence are normally distributed (P-value$<10^{-8}$). The normality test was performed based on the combination of skew test and kurtosis test using scipy (Jones et al. 2011). Whole coding divergence, which is significantly lower than synonymous and average genomic noncoding divergences, but higher than nonsynonymous divergence, was used as threshold. Mean divergence proportion (P) is given by (C-N)/S.

Table S3: Divergence thresholds used

Query	Subject	Whole coding	Skip3	Purpose
Human	Marmoset	0.058 (0.0134)	0.038 (1.239×10^{-3})	Primate common
Mouse	Guinea pig	0.166 (0.0371)	0.095 (1.106×10^{-4})	Rodent common
Dog	Cat	0.082 (0.0024)	0.048 (1.475×10^{-5})	Carnivore common
Cow	Pig	0.098 (0.0008)	0.061 (2.113×10^{-5})	Cetartiodactyl common
Elephant	Human	0.121	0.075	Eutherian ancestral
Elephant	Mouse	0.1652	0.1	Eutherian ancestral
Elephant	Dog	0.126	0.079	Eutherian ancestral
Elephant	Cow	0.132	0.089	Eutherian ancestral
Chicken	Human	0.23	0.144	Tetrapod ancestral
Chicken	Mouse	0.241	0.151	Tetrapod ancestral
Chicken	Dog	0.23	0.146	Tetrapod ancestral
Chicken	Cow	0.234	0.15	Tetrapod ancestral
Chicken	Elephant	0.227	0.145	Tetrapod ancestral
Chicken	Opossum	0.229	0.141	Tetrapod ancestral
Chicken	Platypus	0.226	0.139	Tetrapod ancestral

The values in parentheses are the probabilities of getting alignments of 100bp long and divergence thresholds by chance. The probabilities were calculated using binomial test in R statistical package.

The equation for calculating the probability is given as;

$$\text{Probability} = \sum_{k=0}^{m} \binom{100}{k} x^k (1-x)^{100-k}$$

where m = mismatch threshold (maximum number of mismatches for the threshold);

x = probability of mismatch per site (= genomic noncoding divergence from Table S2).

Table S4: Number of pairwise ancestral CNSs

Query genome	Subject genome	Number of CNSs	
		**Whole coding**	**Skip3**
Chicken	Human	39,857	24,541
	Mouse	28,703	18,544
	Dog	40,778	25,934
	Cow	38,499	25,352
	African elephant	38,700	24,558
	Opossum	37,425	24,525
	Platypus	35,696	22,767
African elephant	Human	311,655	132,503
	Mouse	156,061	54,537
	Dog	277,957	132,423
	Cow	276,173	130,691

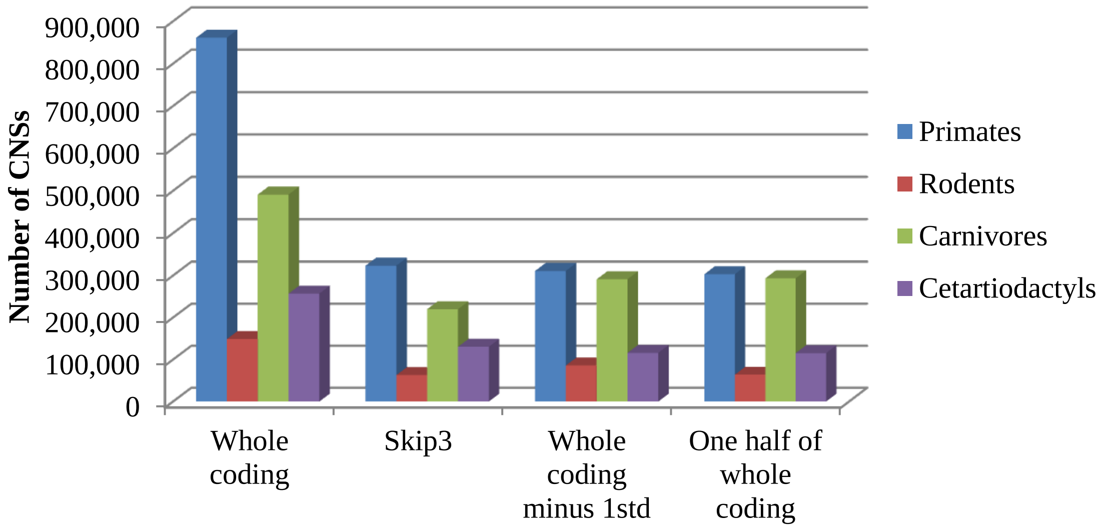

Figure S2: Number of CNSs retrieved with various thresholds. Numbers of CNSs decrease with more stringent thresholds, but the pattern remains essentially the same. We further checked the numbers of CNSs conserved between the reference genomes and the most diverged species of each lineage using threshold divergence minus 1 standard deviation and one half of threshold divergence.

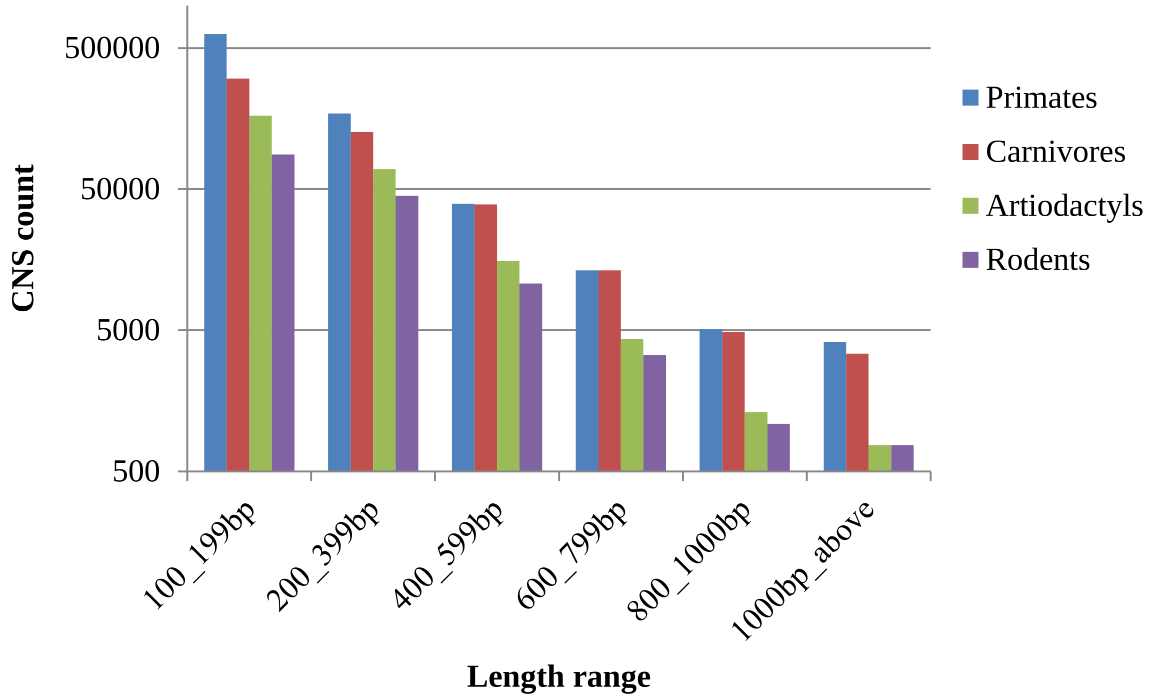

Figure S3: The length distribution of lineage common CNSs. In every length category, primate common CNSs are the most abundant while rodent common CNSs are the least abundant.

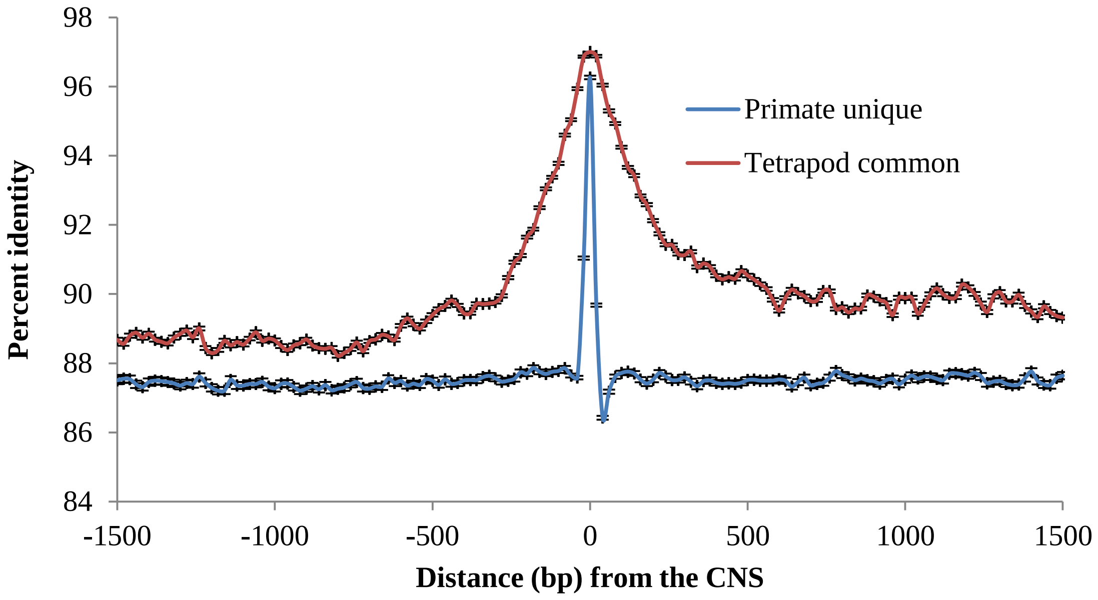

Figure S4: The percent identity of the flanking region of CNS using skip3 threshold. The conservation levels of flanking regions of CNSs with whole coding thresholds. Point 0 is the average percent identity of 100bp at the center of the CNSs while other points are the average of 50bp windows moved at 20bp steps starting from 30pb inside the CNSs. The bars are the standard error of the mean for each window.

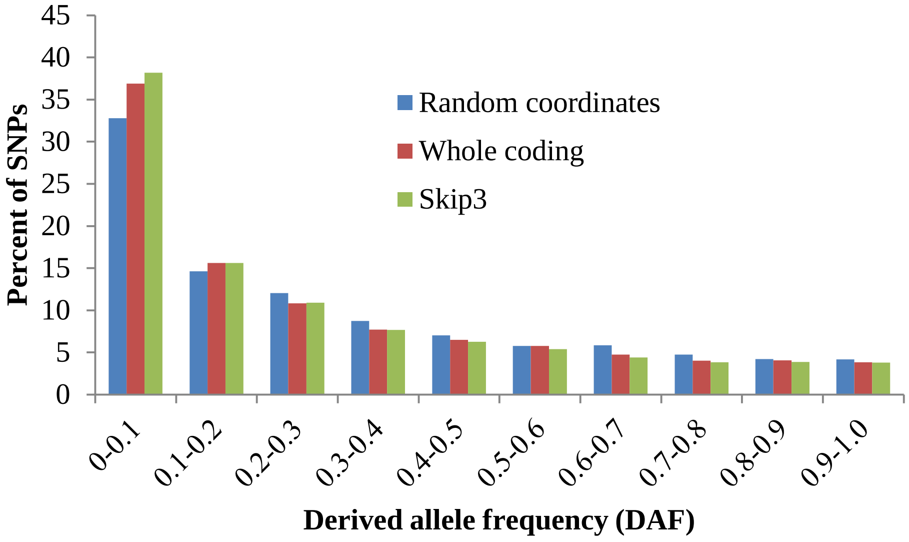

Figure S5: Derived allele frequency analysis for primate unique CNSs using Yoruba population of Hapmap Project III. Compared to the random sequences, most of the SNPs in primate unique CNSs are of lower frequency. At derived allele frequency of 0-0.1, CNSs have significantly higher prortion of SNPs compared to the random sequences (binomial p value $< 10^{-15}$). This suggests that SNPs on CNSs do not spread in the population.

Table S5: Maximum Composite Likelihood Estimate of the Pattern of Nucleotide Substitution

	A	T	C	G
**A**	-	*4.57(4.54)*	*2.76(3.43)*	**13.38(14.65)**
**T**	*4.55(4.53)*	-	**13.32(14.69)**	*2.78(3.44)*
**C**	*4.55(4.53)*	**22.09(19.46)**	-	*2.78(3.44)*
**G**	**21.9(19.33)**	*4.57(4.54)*	*2.76(3.43)*	-

Each entry shows the probability of substitution (r) from one base (row) to another base (column) for tetrapod common CNSs and primate unique CNSs (in parentheses) using whole coding threshold. For simplicity, the sum of r values is made equal to 100. Rates of different transitional substitutions are shown in **bold** and those of transversional substitutions are shown in *italics*. The analysis involved concatenated CNSs of five primates species used. All positions containing gaps and missing data were eliminated. Evolutionary analyses were conducted in MEGA5. GC→AT substitutions (53.11% and 47.86% for tetrapod common and primate unique CNSs, respectively) are higher than AT→GC substitutions (32.24% and 36.21% for tetrapod common and primate unique CNSs, respectively).

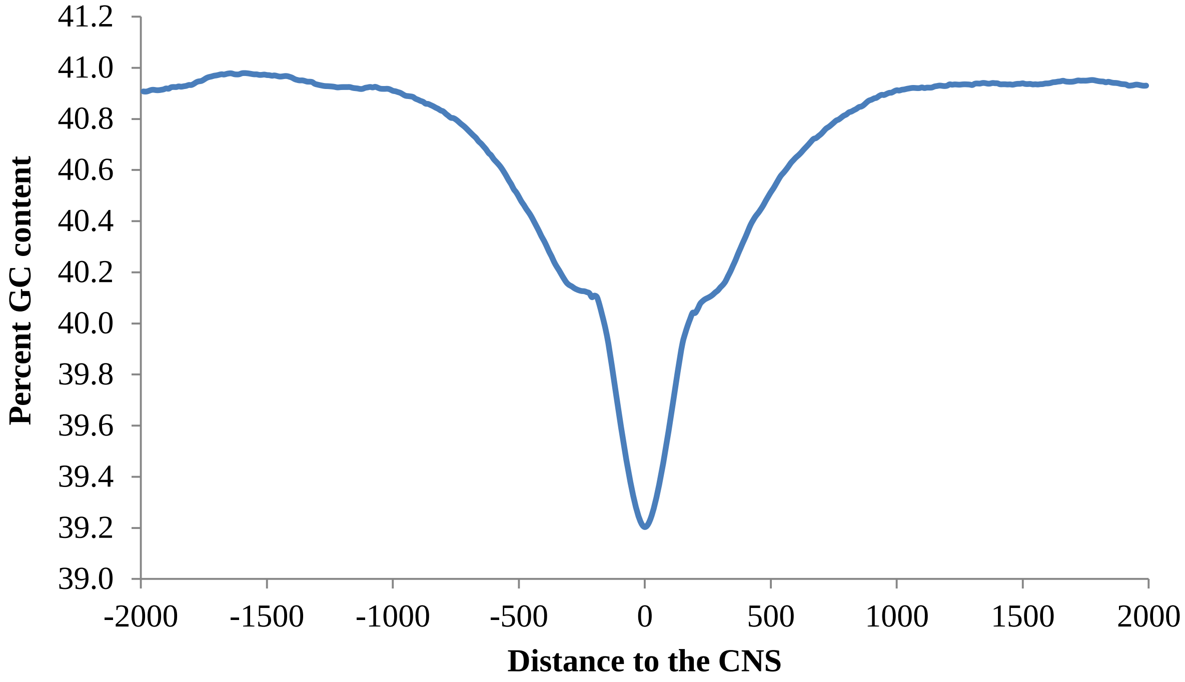

Figure S6: The GC content of the flanking regions of CNSs. Using sliding windows of 200bp size and sliding steps of 10bp, the percent GC contents of the skip3 CNSs and flanking regions were computed. Position 0 is the 100bp in the center of the CNSs and the first window starts from 50bp into the CNSs.

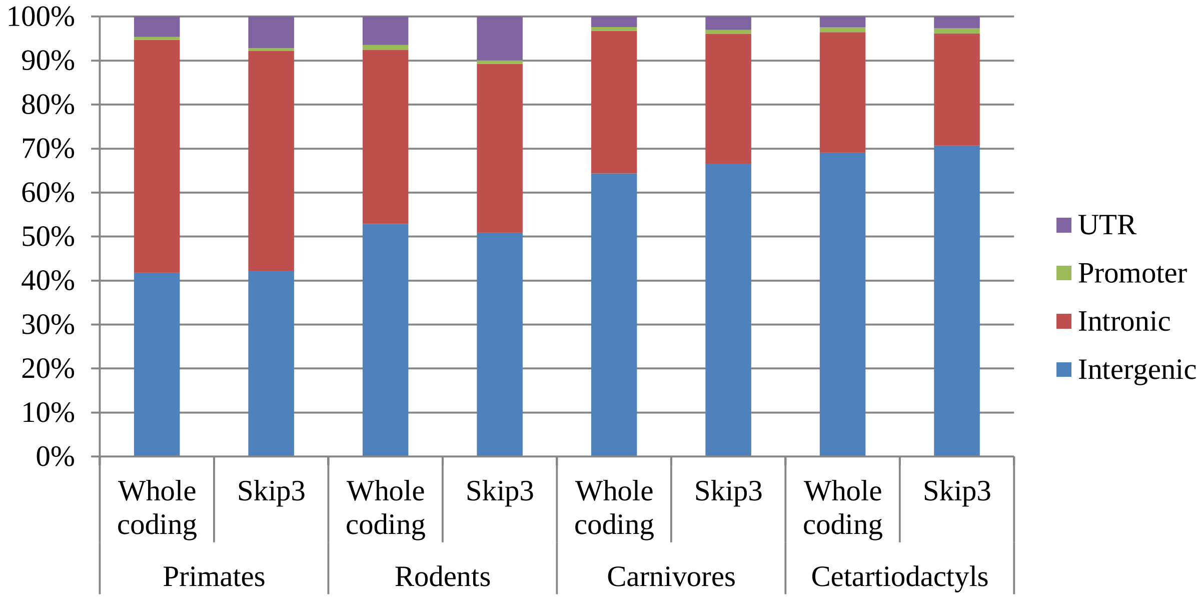

Figure S7: The genomic distribution of lineage common CNSs. The distributions of each lineage are similar in the two thresholds.

Table S6: The genomic locations of the orthologous eutherian common CNSs between pairs of species

**Species**		**Total**												
**Mouse**	**Inter**	**18342**	**16582**	1551	39	170								
	**Intr**	**15209**	410	**14467**	12	320								
	**Prom**	**197**	32	32	**92**	41								
	**UTR**	**2158**	101	154	21	**1882**								
**Dog**	**Inter**	**22785**	**16861**	4910	71	943	**17895**	3926	86	878				
	**Intr**	**11885**	201	**11172**	11	501	352	**11166**	17	350				
	**Prom**	**259**	15	70	**59**	115	20	71	**66**	102				
	**UTR**	**977**	48	52	23	**854**	75	46	28	**828**				
**Cow**	**Inter**	**24807**	**17068**	6390	85	1264	**18189**	5353	90	1175	**21408**	2803	64	532
	**Intr**	**10122**	44	**9712**	2	364	131	**9750**	4	237	1108	**8938**	33	43
	**Prom**	**292**	7	87	**76**	122	11	83	**99**	99	43	62	**123**	64
	**UTR**	**685**	6	15	1	**663**	11	23	4	**647**	226	82	39	**338**
		Total	**17125**	**16204**	**164**	**2413**	**18342**	**15209**	**197**	**2158**	**22785**	**11885**	**259**	**977**
			**Inter**	**Intr**	**Prom**	**UTR**	**Inter**	**Intr**	**Prom**	**UTR**	**Inter**	**Intr**	**Prom**	**UTR**
		Species	**Human**				**Mouse**				**Dog**			

Inter - intergenic; Intr - intronic; Prom - promoter; UTR - untraslated region

The numbers in grey background represent the values of orthologous CNSs located on the same genomic locations genomic locations between the pair of species. Although for all pairs considered majority of the CNSs are located in homologous regions, some are located in different regions. For this analysis, whole coding threshold was used.

Table S7: Genomic location with Skip3 thresholds

	Human	Mouse	Dog	Cow
**Human**		1,024 (7.73%)	2,590 (19.56%)	3,170 (23.94%)
**Mouse**	12,215		2,212 (16.71%)	2,798 (21.14%)
**Dog**	10,649	11,027		1,781 (13.45%)
**Cow**	10,069	10,441	11,456	

Using Skip3 thresholds, genomic locations of the 13,239 single-copy CNSs that are shared by all eutherian species used were compared in the four representative species. The numbers in grey shade are located on the same genomic location while the numbers in the upper part (without shade) are located on different locations.

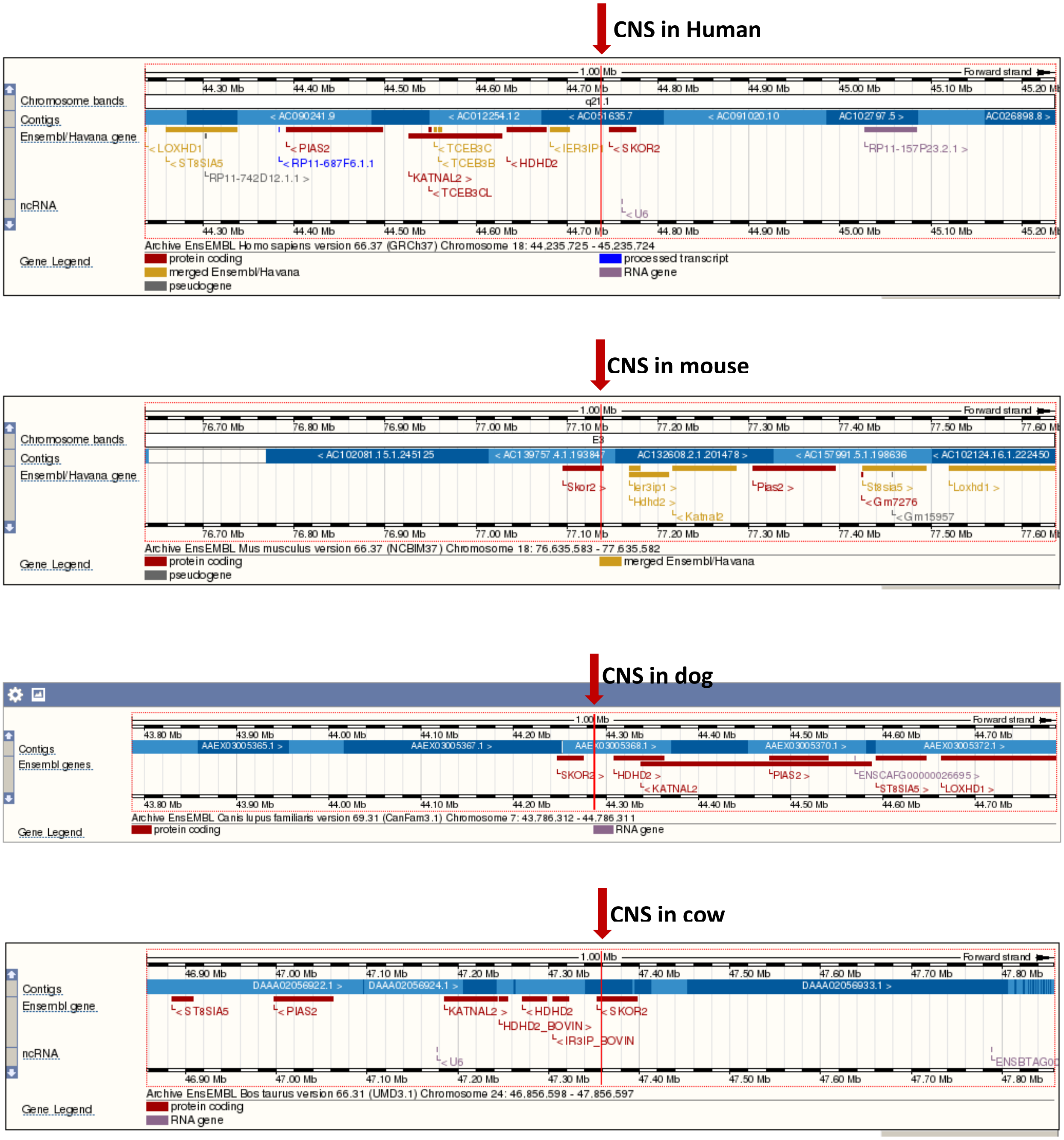

Figure S8: An example of CNS located on different genomic region. The position of the CNS is represented by the red vertical line at the center of each representation. In cow and mouse, the CNS is located in the intron of SKOR2 gene but in human and dog, it is found in the intergenic region outside SKORE2 gene.

(a)

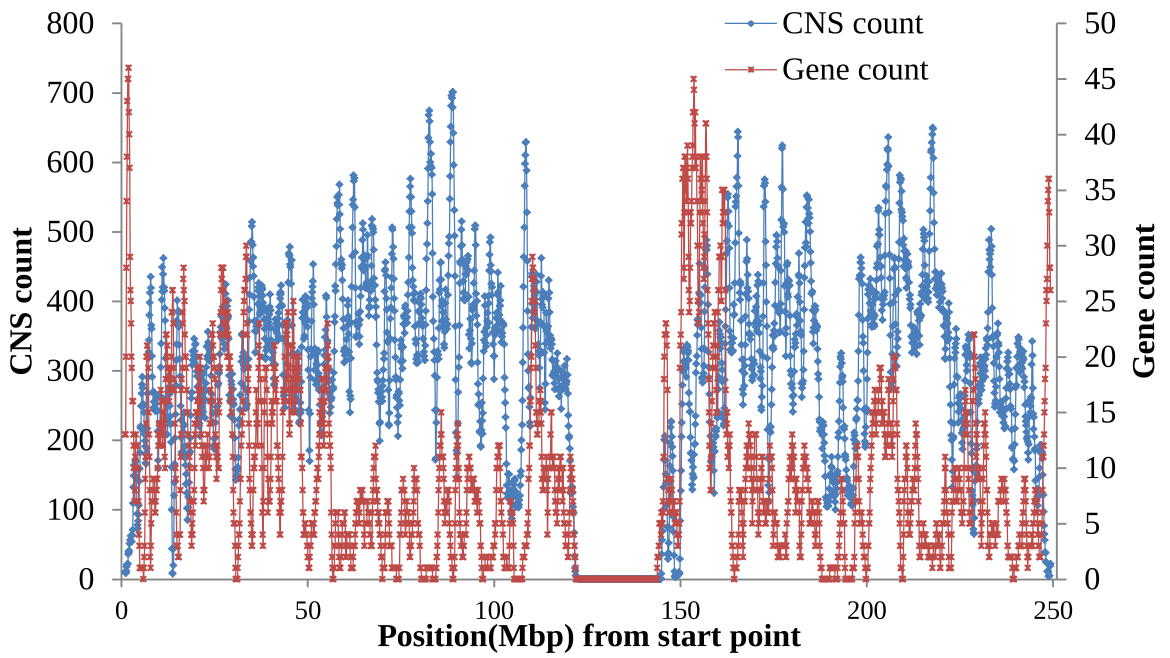

(b)

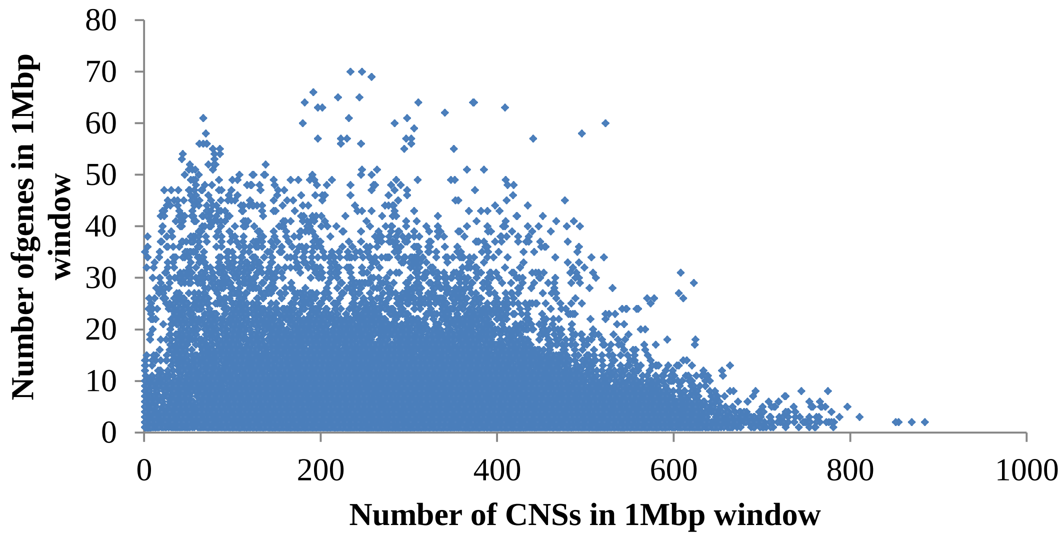

Figure S9: Distribution of CNSs and genes. (a) Distribution on human chromosome 1. CNSs and genes are not found around the centromeres and there distribution on the chromosome is not uniform. Each point represents 1Mbp window and the window was moved at 100kbp per step. (b) Distribution of all windows with at least a CNS and a gene. Pearson's r =-0.2234 (P-value<8.069×10^{-299}).

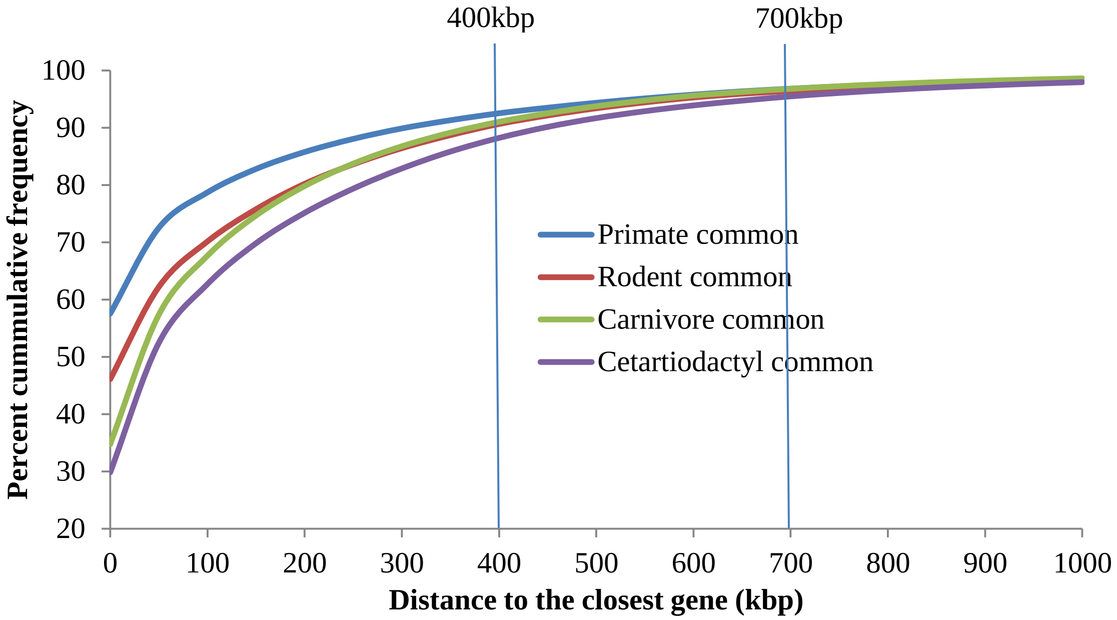

Figure S10: The proximity of the CNSs to genes. The horizontal axis represents the distance of the CNS to the closest protein coding gene using whole coding divergence.

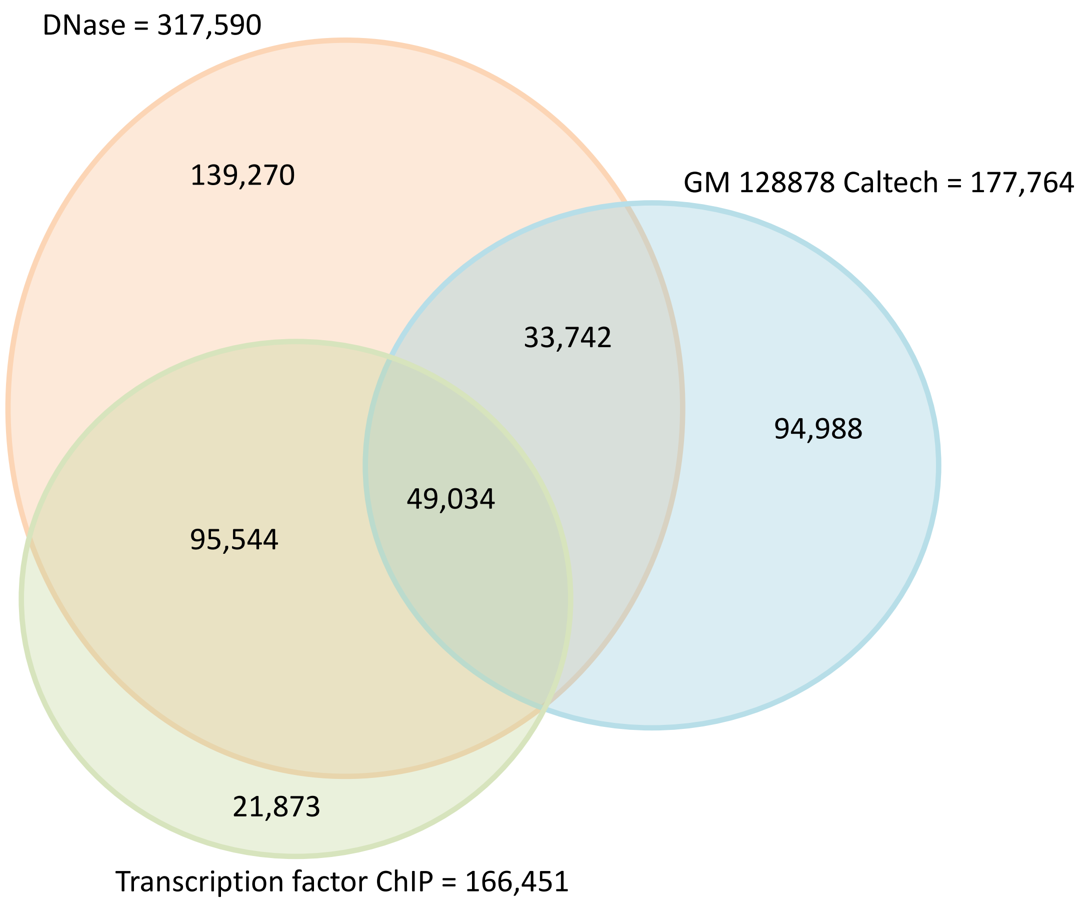

Figure S11: Overlap of primate common CNSs with selected regulatory signatures. The numbers of primate common CNSs that overlap three regulatory signatures are shown. In total, 434,451 CNSs overlap with at least one of the three regulatory signatures. GM128878 Caltech are the data of GM128878 cell line from ENCODE project while the transcription factor ChIP are the transcription factor binding site clusters. Also, overlap with DNase was checked.

(a)

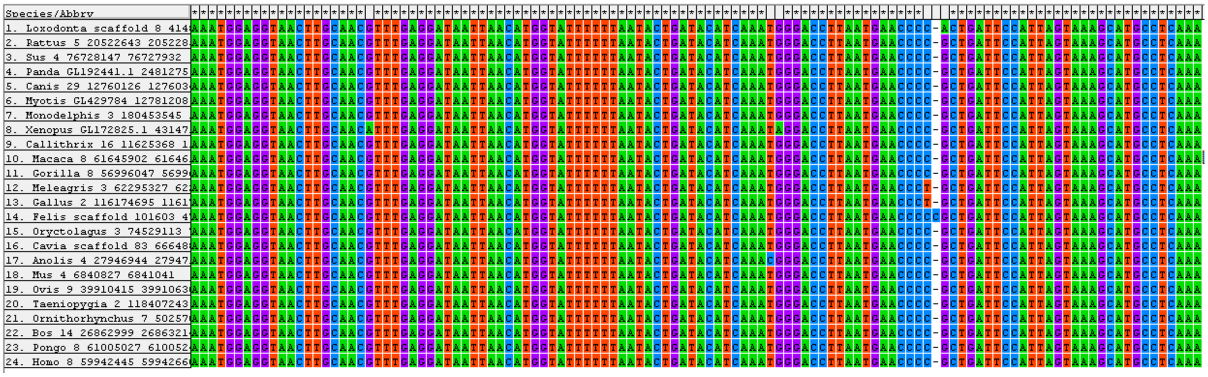

(b)

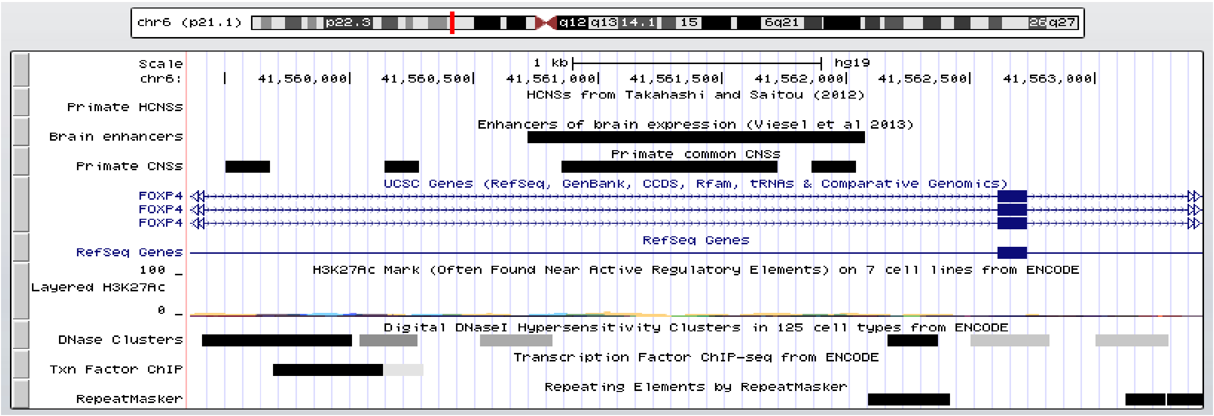

(c)

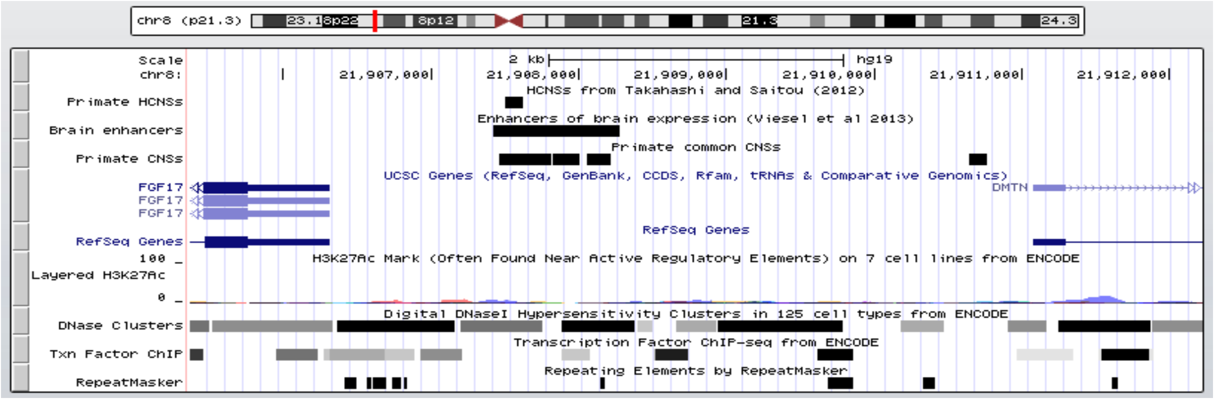

Figure S12: Examples of identified CNSs. (a) An example of the multiple alignement of CNS showing high sequence conservation. (b) Four syntenic primate common CNSs with two overlapping brain enhancer, two overlapping DNase clusters and one overlapping transcription factor. (c) Four identified primate common CNSs located in intergenic region. Three overlap brain enhancer and one has no annotated regulatory signature. The CNSs shown were identified using whole coding thresholds.

Table S8: The gene ontology analysis using binomial test as described by PANTHER

Biological process	Tetrapod common	Primate unique	Rodent unique	Carnivore unique	Cetartiodactyl unique	Mouse-lost
**Transcription**	7.44E-108	0.046277	1.13E-27	0.414611	0.010728	9.37E-244
**Development**	3.18E-119	0	9.95E-20	3.10E-08	3.44E-05	0
**Nervous system**	3.58E-48	2.57E-300	3.77E-12	8.05E-06	0.405432	5.59E-134
**Response to stimulus**	3.64E-07	4.51E-180	0.024622	0.0811	0.406716	1.32E-44
**Immune and defense**	1.76E-13	3.48E-83	0.215629	0.698506	0.00055	7.23E-45

The values in gray shade are significant (Binomial P-value<0.001). Transcription, development and nervous system related genes are overrepresented while response to stimulus, immune and defense related genes are underrepresented. Genes under negative selection are more associated with CNSs while genes under positive selection are less associated with selective constraint.

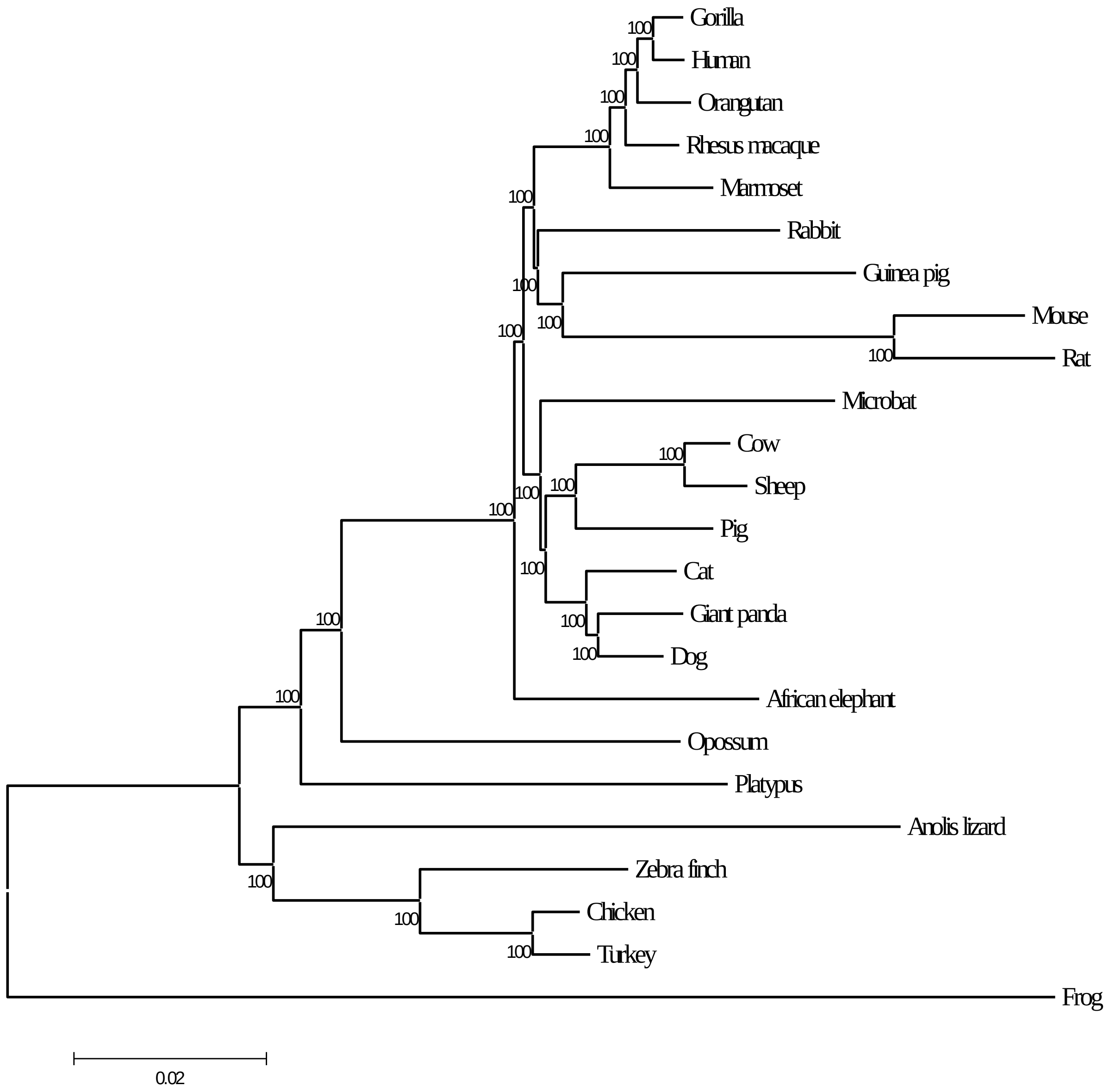

Figure S13: The phylogenetic tree of the CNSs. The concantenated tetrapod common CNSs, using whole coding thresholds, were used to construct the phylogenetic tree using NJ method. All branches were supported with 100% boolstrap values.
